# The *NCF1* variant p.R90H aggravates autoimmunity by facilitating the activation of plasmacytoid dendritic cells

**DOI:** 10.1172/JCI153619

**Published:** 2022-08-15

**Authors:** Yao Meng, Jianyang Ma, Chao Yao, Zhizhong Ye, Huihua Ding, Can Liu, Jun Li, Guanhua Li, Yuke He, Jia Li, Zhihua Yin, Li Wu, Haibo Zhou, Nan Shen

**Affiliations:** 1 Shanghai Institute of Rheumatology, Renji Hospital, School of Medicine, Shanghai Jiao Tong University (SJTUSM), Shanghai, China.; 2 Shenzhen Futian Hospital for Rheumatic Diseases, Shenzhen, China.; 3 China Australia Centre for Personalized Immunology, Renji Hospital, School of Medicine, Shanghai Jiao Tong University (SJTUSM), Shanghai, China.; 4 Tsinghua-Peking Joint Center for Life Sciences, Tsinghua University School of Medicine, Beijing, China.; 5 Center for Autoimmune Genomics and Etiology (CAGE), Cincinnati Children’s Hospital Medical Center, Cincinnati, Ohio, USA.; 6 Department of Pediatrics, University of Cincinnati College of Medicine, Cincinnati, Ohio, USA.; 7 State Key Laboratory of Oncogenes and Related Genes, Shanghai Cancer Institute, Renji Hospital, Shanghai Jiao Tong University School of Medicine (SJTUSM), Shanghai, China.

**Keywords:** Autoimmunity, Lupus

## Abstract

Plasmacytoid dendritic cells (pDCs) are a professional type I IFN producer that play critical roles in the pathogenesis of autoimmune diseases. However, both genetic regulation of the function of pDCs and their relationships with autoimmunity are largely undetermined. Here, we investigated the causality of the neutrophil cytosolic factor 1 (*NCF1*) missense variant, which is one of the most significant associated risk variants for lupus, and found that the substitution of arginine (R) for histidine (H) at position 90 in the NCF1 protein (NCF1 p.R90H) led to excessive activation of pDCs. A mechanism study demonstrated that p.R90H reduced the affinity of NCF1 for phospholipids, thereby impairing endosomal localization of NCF1. As NCF1 is a subunit of the NADPH oxidase 2 (NOX2) complex, this impairment led to an acidified endosomal pH and facilitated downstream TLR signaling. Consistently, the homozygous knockin mice manifested aggravated lupus progression in a pDC-dependent lupus model. More important, pharmaceutical intervention revealed that hydroxychloroquine (HCQ) could antagonize the detrimental function of NCF1 p.R90H in the lupus model and systemic lupus erythematosus samples, supporting the idea that NCF1 p.R90H could be identified as a genetic biomarker for HCQ application. Therefore, our study provides insights into the genetic control of pDC function and a paradigm for applying genetic variants to improve targeted therapy for autoimmune diseases.

## Introduction

Genetic evidence and clinical studies have revealed that the type I interferon (IFN-I) pathway is a primary pathogenic factor in a variety of autoimmune diseases (1–3). As the major IFN-I–producing cell subset, plasmacytoid dendritic cells (pDCs) were thought to play a predominant role in the occurrence and progression of autoimmunity (4). GWAS have attempted to elucidate the complex risk variants across different populations. Despite the growing number of reported common, rare, and de novo mutations, few studies have focused on surveying the functional impact of genetic variants on pDCs.

PDCs are crucial mediators of innate immunity (4, 5). When challenged with autoantigens, pDCs were aberrantly activated, producing excessive amounts of IFN-I and other proinflammatory cytokines to facilitate Th1 differentiation and autoreactive B cell expansion (5). pDCs sense nucleic acids via TLR7 and TLR9, which shuttle from the endoplasmic reticulum to the endosome via vesicles from the Golgi. Acidification of the endosome and proteolytic cleavage of TLR7 and TLR9 are essential for TLR signaling in response to ligand binding (6–10). The endosomal pH is controlled by the respective activities of 2 large molecular complexes: the v-ATPase imports protons across the endosomal membrane, and NADPH oxidase 2 (NOX2) causes proton consumption (11–14). Depending on the recruitment and activity of these 2 complexes, the pH acidifies or alkalinizes.

To find the critical genetic variant that most likely regulates the function of pDCs, we searched for the strongest loci associated with systemic lupus erythematosus (SLE) and other IFN-I–related autoimmune diseases deduced from Immunochip data. We identified what we believe to be a novel missense SNP, rs201802880, in neutrophil cytosolic factor 1 (*NCF1*), also known as *p47^phox^*, that was strongly associated with SLE (OR = 3.47 and minor allele frequency [MAF] = 0.3985 in Koreans and Chinese (*P*_meta_ = 3.1 × 10^−104^); OR = 2.61 and MAF = 0.0575 in White people of European descent; OR = 2.02 and MAF = 0.1570 in African Americans) and other autoimmune diseases, including primary Sjögren’s syndrome (SS) (OR = 2.45 in Chinese, OR = 2.35 in White people of European descent) and rheumatoid arthritis (RA) (OR = 1.65 in Koreans) (15). SLE is an autoimmune disease characterized by autoantibodies against nuclear and cytoplasmic antigens, as well as systemic inflammation affecting skin, kidneys, and other organs. The substitution from arginine (R) to histidine (H) at position 90 in NCF1 protein (NCF1 p.R90H) was verified to parallel with lupus-related symptoms, including altered formation of neutrophil extracellular traps (NETs), a high IFN-I signature, and antiphospholipid syndrome (APS) (16–18). NCF1 encodes the key regulatory component of the phagocytic NADPH oxidase 2 (NOX2) complex, which generates ROS (19). Hence, it suggested that NCF1 p.R90H might be involved in the pathogenesis of autoimmune diseases through disturbance of ROS homeostasis in pDCs and alteration of IFN-I production.

In this study, we explored the function of NCF1 p.R90H in lupus by using knockin (KI) mice. The R-to-H change in NCF1 led to decreased phospholipid-binding affinity, less endosomal localization, and acidified endosomal pH, followed by increased cleavage of TLR7 and TLR9, resulting in excessive activation of pDCs and aggravated lupus progression. More important, our study of the therapeutic effect of hydroxychloroquine (HCQ) on KI mice identified a genetic biomarker for improving the treatment of SLE.

## Results

### NCF1 p.R90H mutation facilitates the activation of pDCs.

**Figure 1:**
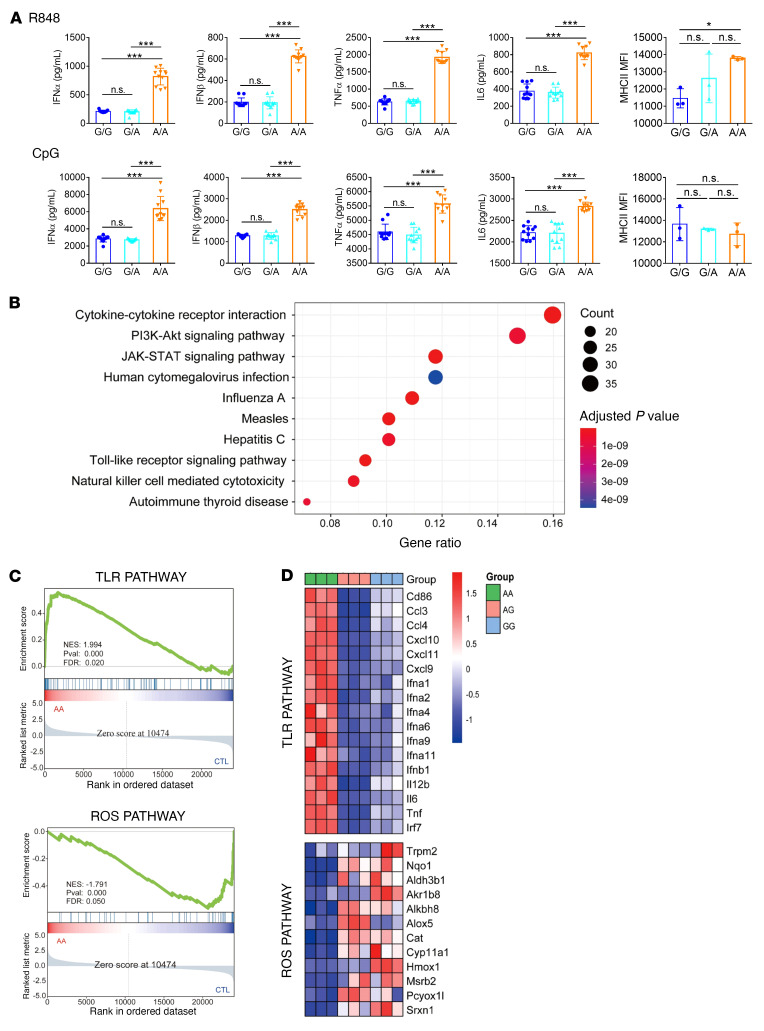
The NCF1 p. R90H mutation facilitates pDC activation. (**A**) Splenic pDCs from WT (G/G), homozygous (A/A), and heterozygous (G/A) KI mice were sorted and then stimulated with R848 (upper panel) or CpG (lower panel) for 24 hours. Expression of IFN-α, IFN-β, TNF-α, and IL-6 and MHCII levels are shown. Error bars represent the SEM. *n =* 12. *P < 0.05 and ***P < 0.001, by 1-way ANOVA with Tukey’s multiple-comparison test. (**B**–**D**) Sorted splenic pDCs with a specific genotype (G/G, G/A, and A/A) were stimulated with R848 for 12 hours and subjected to RNA-Seq. (**B**) KEGG analysis of upregulated genes in A/A compared with G/G and G/A pDCs. (**C**) GSEA analysis of TLR pathway and ROS pathway enrichment in A/A and control (CTL) (G/G and G/A) pDCs. NES, normalized enrichment score; Pval, *P* value.(**D**) Heatmap of changed genes enriched in TLR and ROS pathways.

As NCF1 p.R90H is strongly associated with an elevated IFN-I signature in SLE, it drew our attention to investigating the function of this SNP in pDCs. It was impossible to edit human primary pDCs directly, so we turned to constructing the *Ncf1*-KI mice to obtain pDCs with different alleles (Supplemental Figure 1; supplemental material available online with this article; https://doi.org/10.1172/JCI153619DS1). The base changed from G to A, corresponding to the amino acids from R to H. Splenic pDCs were isolated from WT (G/G), homozygous (A/A), and heterozygous (G/A) KI mice. Then, pDCs were stimulated with the TLR7/-8 ligand R848 and the TLR9 ligand CpG, respectively. As shown in Figure 1A, compared with G/G pDCs, the production of IFN-α and IFN-β was enhanced in A/A pDCs, with a 3-fold increase in the case of R848 stimulation and a 2-fold in the case of CpG stimulation (Figure 1A). In contrast, we observed no significant difference between G/A and G/G pDCs. Similarly, we detected elevated expression of proinflammatory cytokines including TNF-α and IL-6 and of MCH class II (MHCII) in A/A pDCs, whereas expression levels in G/A pDCs were almost the same as those in the control G/G pDCs (Figure 1A). Thus, NCF1 p.R90H contributed to the overactivation of pDCs following TLR stimulation.

### NCF1 p.R90H mutation augments the TLR signaling pathway.

To get a whole picture of its role in pDC activation, we performed RNA profiling of pDCs with or without the NCF1 p.R90H variant. We stimulated pDCs of a specific genotype (G/G, G/A, or A/A) with R848 for 12 hours and then subjected them to RNA-Seq. Upregulated genes in A/A pDCs were mainly enriched in the TLR pathway, the PI3K/AKT pathway, and the JAK/STAT pathway (Figure 1B). Gene set enrichment analysis (GSEA) confirmed the enhanced TLR activation in A/A pDCs compared with G/A and G/G pDCs (Figure 1C). Interestingly, ROS-related genes were downregulated in A/A pDCs, consistent with the previous finding that NCF1 participates in the regulation of ROS production. Specifically, compared with G/G and G/A pDCs, IFN-related genes such as IFN-I subtypes and transcriptional factor IFN regulatory factor 7 (IRF7) were expressed at higher levels in A/A pDCs. Furthermore, we found that the expression of proinflammatory cytokines was also augmented by the NCF1 p.R90H variant, comprising *Tnf*, *Il6*, and *Il12b*, chemokines such as C-C motif chemokine ligand 3 *(Ccl3*), *Ccl4*, C-X-C motif chemokine ligand 9 *(Cxcl)*, *Cxcl10*, and *Cxcl11*, and the costimulatory molecule *Cd86* (Figure 1D, upper panel). Meanwhile, redox-responsive genes, including transient receptor potential cation channel subfamily M member *(Trpm) 2* and catalase (*Cat*), and genes responsible for ROS generation, such as NAD(P)H quinone dehydrogenase *(Nqo1*) and heme oxygenase *(Hmox1*), were found to be downregulated in A/A pDCs (Figure 1D, lower panel). Moreover, the phosphorylation levels of IRF7 and NF-κB increased in A/A pDCs under the stimulation of R848 or CpG (Supplemental Figure 2A). Taken together, RNA profiling indicated that NCF1 p.R90H led to excessive activation of the TLR pathway and less oxidative stress in pDCs.

### NCF1 p.R90H impairs endosomal localization of NCF1 and acidifies endosomal pH.

Considering the extensive effect of NCF1 p.R90H on pDC activation, we next studied the mechanism. As NCF1 p.R90H contributed to dysregulation of the ROS pathway (Figure 1, C and D), we examined the ROS change in pDCs. We found that A/A pDCs had lower ROS levels than did G/A or G/G pDCs (Supplemental Figure 2B). To further discriminate the origination of these divergent ROS levels, we measured mitochondrial ROS levels in pDCs using MitoSOX Red, a fluorogenic dye for highly selective detection of superoxide in the mitochondria of live cells. We found that mitochondrial ROS levels of A/A pDCs were similar to those of G/G pDCs (Supplemental Figure 2C). Thus, the difference in ROS levels in G/G and A/A pDCs is more likely to be derived from NOX2. ROS have been proven to influence the metabolic state of cells including glycolysis, oxidative phosphorylation (OXPHOS), fatty acid oxidation (FAO), and redox homeostasis (20). To identify the specific metabolic process involved in the regulation of pDC function, etomoxir (FAO inhibitor), 2-deoxyglucose (2-DG, glycolysis inhibitor), metformin (OXPHOS inhibitor) or *N*-acetyl-l-cysteine (NAC, ROS inhibitor) were added to the culture medium and pDC cytokine production was measured. Interruption of the energy supply fueled by FAO, glycolysis, or OXPHOS suppressed the production of IFN-I by pDCs, while ROS clearance significantly boosted pDC IFN-I generation (Supplemental Figure 3, A and B). Of note, compared with other inhibitors, only NAC pretreatment eliminated the difference in the production of IFN-I between A/A and G/G pDCs. Furthermore, overexpression of the antioxidant enzyme catalase had an effect similar to that seen with NAC treatment, indicating the critical role of ROS in the regulation of pDC function by NCF1 p.R90H (Supplemental Figure 3, C and D).

**Figure 2:**
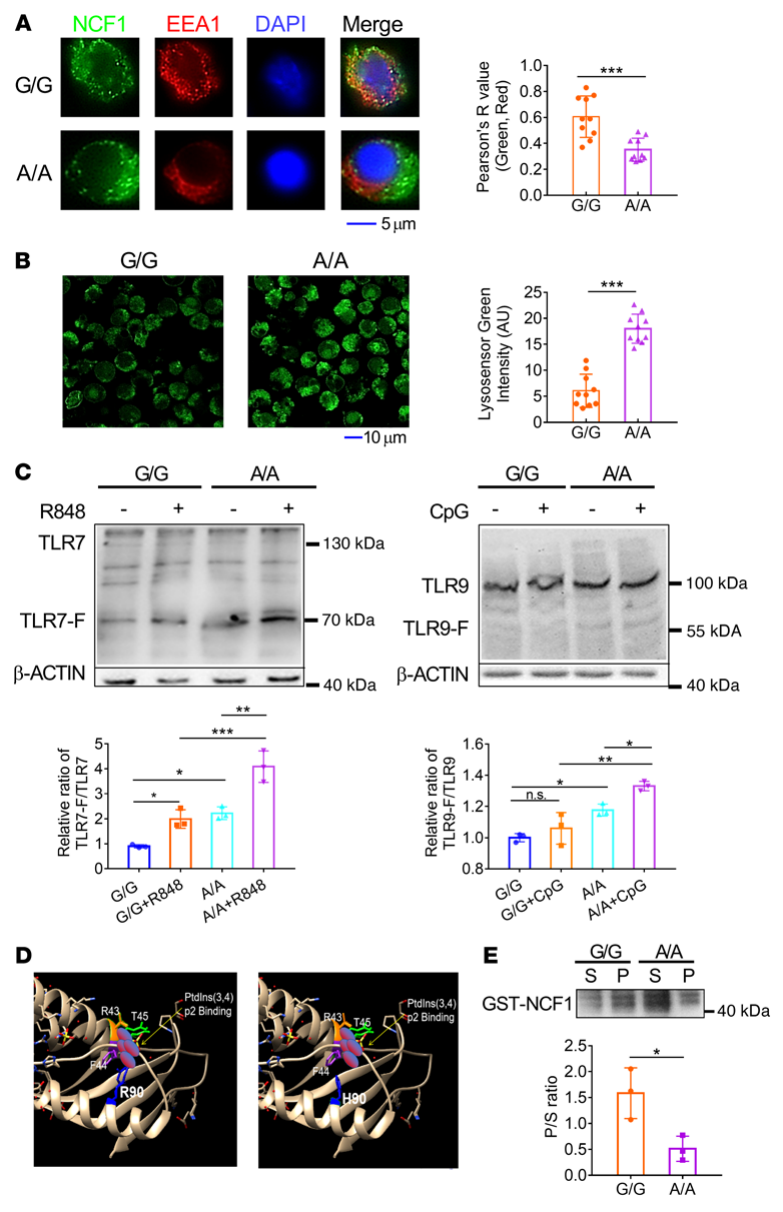
NCF1 p.R90H impairs endosomal localization of NCF1 and acidifies the endosomal pH. pDCs were stimulated with CpG for 2 hours. (**A**) Colocalization of NCF1 (green) and EEA1 (red) was detected by confocal microscopy and analyzed by ImageJ. Scale bar: 5 μm. One data point in the plot indicates the mean of Pearson’s *R* values from 5 cells, and a total of 50 cells per group were calculated . (**B**) The pH of late endosomes/lysosomes in CpG-activated pDCs was indicated by a lysosome sensor, detected by confocal microscopy, and measured by ImageJ. Scale bar: 10 μm. One data point in the plot indicates the average MFI of 5 cells, and a total of 50 cells per group were calculated. (**C**) Immunoblot analysis of cleavage of TLR7 and TLR9. G/G and A/A pDCs were stimulated with R848 or 5 μM CpG for 1 hour, respectively. The levels of intact TLR7 and the cleaved TLR7 fragment (TLR7-F) in R848-treated groups and the levels of intact TLR9 and the cleaved TLR9 fragment (TLR9-F) in the CpG-treated groups were analyzed. The ratios of cleaved TLR to intact TLR were calculated. (**D**) Structure of the PX domain of NCF1 90R and 90H. R90 and R43 are amino acids responsible for PtdIns(3,4)p2 binding. (**E**) Immunoblot analysis of GST-NCF1. S, protein in supernatant, P, protein in precipitation. The P/S ratios were calculated. Experiments were repeated 3 times. Error bars represent the SEM. *P < 0.05, **P < 0.01, and ***P < 0.001, by 2-tailed, unpaired *t* test (**A**, **B**, and **E**) or 1-way ANOVA with Tukey’s multiple-comparison test (**C**).

The activation of pDCs involves the production of IFN-I by the TLR-driven PI3K/IRF7 pathway and the generation of proinflammatory factors, chemokines, and costimulatory molecules governed by the TLR-activated NF-κB pathway (4). The overall enhancement of pDC function caused by the *Ncf1* variant prompted us to speculate that the initial step of the TLR pathway was affected. TLR7/-8 and TLR9 recognize and bind to ligands in the endosome. Given that ROS also engage in endosomal regulation, we next investigated whether NCF1 p.R90H affected endosomal function. First, we confirmed that both the protein and phosphorylation levels of NCF1 were not affected after TLR stimulation (Supplemental Figure 4A). In contrast, the colocalization of NCF1 with the endosome marker early endosome antigen 1 (EEA1) was found to be decreased, suggesting impaired translocalization of NCF1 to the endosome (Figure 2A). Correspondingly, the pH values of late endosomes/lysosomes were lower in A/A pDCs, than in control G/G pDCs (Figure 2B). Heterozygous G/A pDCs had pH levels similar to those of G/G pDCs (Supplemental Figure 4B). In contrast, we found no difference in the colocalization of NCF1 with LAMP1 (a lysosome marker) between A/A and G/G pDCs (Supplemental Figure 4C).

It has been reported that RAC2 is essential for assembly of the NOX2 complex on the endosome membrane (21). We also confirmed that RAC2 was involved in regulating the translocation of NCF1 in pDCs, as knockdown of RAC2 dampened the colocalization of NCF1 with EEA1 (Supplemental Figure 4, D and E). Since both RAC2 and NCF1 are subunits of the NOX2 complex, these results supported the idea that NCF1 functions in the form of a NOX2 complex.

The endosomal pH of DCs is controlled by the respective activities of the v-ATPase complex and the NOX2 complex. In addition, endosomal NOX2–derived ROS have been reported to inhibit the activity of the v-ATPase complex in neutrophils (22). Impaired localization of 90H NCF1 might lead to a lower endosomal pH either by disrupting the generation of ROS that consume protons, or by enhancing the activity of the v-ATPase that pumps more protons into the endosome (23). To confirm whether the v-ATPase complex is involved in this process, we examined the effect of bafilomycin A (a specific inhibitor of v-ATPase) on the activation of pDCs with A/A or G/G alleles (24). After bafilomycin A pretreatment, we found that IFN-α and IFN-β production was markedly decreased. In addition, the difference between A/A and G/G pDCs was eliminated, indicating that v-ATPase mediated the function of NCF1 in this process (Supplemental Figure 4F). Moreover, another voltage-gated proton channel, Hv1, was reported to control TLR9 activation in pDCs by assisting the v-ATPase during immediate endosomal acidification (25). To verify whether the Hv1 complex also participated in the regulation of pDC function, pDCs were subjected to treatment with an Hv1-specific inhibitor, 2-guanidinobenzimidazole (2-GBI) (26, 27). We found that 2-GBI dramatically suppressed the production of type I IFNs by A/A and G/G pDCs (Supplemental Figure 4G). These data suggested that both Hv1 and v-ATPase complexes might be involved in promoting proteolytic cleavage of TLR ligands by facilitating endosomal acidification in pDCs.

An acidic pH is necessary for the proper activity of endosomal acid proteases, which cleave TLR receptors before their recognition of and binding to ligands (14). To evaluate the effect of pH acidification by NCF1 p.R90H in A/A pDCs, we measured both integrated and cleaved TLRs. Compared with control G/G pDCs, A/A pDCs had an increased ratio of cleaved TLRs to intact TLRs (Figure 2C).

Given that NCF1 p.R90H caused the defective localization of NCF1 on the endocytic membrane, we next explored the reasons. The arginine at position 90 is considered to be important for NCF1 binding of phosphatidyl inositol 3,4-bisphosphate [PtdIns(3,4)P2], according to the structure resolution (28, 29). It is probable that this variant might change the affinity of NCF1 A/A or G/G for phospholipids. To verify our hypothesis, we analyzed the structural change caused by amino acid variation. According to a previous report, R43 and R90 are in the region of the phosphoinositide-binding pocket and critical for PtdIns(3,4)P2 binding (28). The variant from R to H led to a conformation alteration, wehreby H left the binding pocket (Figure 2D). Then, we incubated synthetic phospholipid vesicles with purified glutathione S-transferase–tagged (GST-tagged) NCF1 A/A or G/G protein in vitro. Phospholipid binding protein was centrifuged into the precipitate, while unbound protein was retained in the supernatant. The relative ratio of proteins in the precipitate to those in the supernatant indicates the binding affinity. We found that the p.R90H mutation dramatically reduced the ability of NCF1 to bind to phospholipid and PtdIns(3,4)P2 (Figure 2E).

In summary, NCF1 p.R90H resulted in impaired localization of NCF1 at the endosome, followed by lowered pH and more cleavage of TLRs, which resulted in overactivation of the TLR signaling pathway.

### Ncf1-KI mice exhibit aggravated lupus progression.

**Figure 3:**
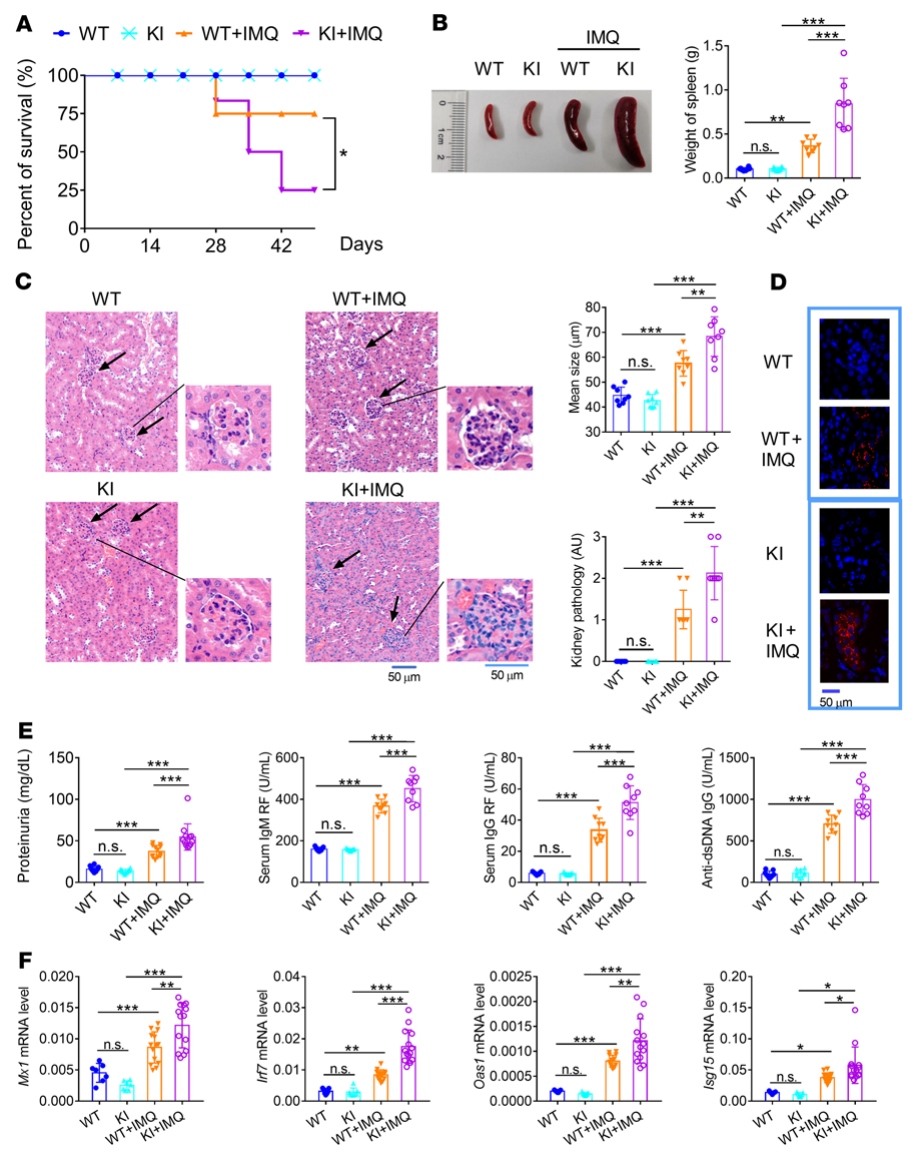
*Ncf1*-KI mice exhibit aggravated lupus progression. WT and KI mice were treated with IMQ for 9 weeks. (**A**) Survival of WT mice (WT), KI mice (KI), WT lupus mice (WT+IMQ) and KI lupus mice (KI+IMQ). *n* = 20 mice per group. (**B**) Image and weight of spleens (*n =* 8). (**C**) H&E staining, glomerulus size, and pathological scores for renal tissues. Scale bars: 50 μm. (**D**) Deposition of anti-IgG antibody in the glomerulus (blue, DAPI; red, Alexa Fluor 647 goat anti–mouse IgG antibody). (**E**) Levels of proteinuria (*n =* 12), serum IgM and IgG RF, and anti–dsDNA IgG (*n =* 9). (**F**) MRNA levels of *Mx1*, *Irf7*, *Oas1*, and *Isg15* in renal tissues (*n =* 7 or 14). The expression of mRNAs was normalized to the housekeeping gene *Actb* mRNA by calculating 2^–ΔCt^. Error bars represent the SEM. *P < 0.05, **P < 0.01, and ***P < 0.001, by 1-way ANOVA with Tukey’s multiple-comparison test.

After elucidating the regulation of NCF1 p.R90H in pDCs in vitro, we sought to clarify its effect on lupus in vivo. No abnormalities were observed in KI mice with regard to the percentage of different immune cell subpopulations prior to the imiquimod (IMQ) challenge. Strategically, both WT and KI mice were subjected to IMQ to establish the lupus model, which has been proven to be dependent on the production of IFN-I and IFN-III in pDCs (30, 31). The overall survival rate for KI lupus mice was lower than that for WT lupus mice (Figure 3A). Both splenomegaly and splenic weight increased in the KI lupus mice (Figure 3B). H&E staining of renal tissues indicated that the KI mice developed more serious pathological features, including larger glomeruli and more infiltration of immune cells (Figure 3C). A greater deposition of IgG antibodies was observed in the kidneys of KI mice (Figure 3D). Consistent with this, the levels of proteinuria and autoantibodies, including rheumatoid factors (RFs) and anti-dsDNA antibodies in serum, were all elevated in KI lupus mice (Figure 3E). Meanwhile, the expression levels of IFN-I signature genes (ISGs), such as *Mx1*, *Irf7*, *Oas1*, and *Isg15*, were increased in the renal tissues of KI lupus mice compared with WT lupus mice (Figure 3F). Thus, we confirmed that NCF1 p.R90H aggravated the lupus progression. Additionally, the G/A heterozygous mice showed phenotypic changes similar to those of control WT mice across IMQ applications (data not shown).

**Figure 4:**
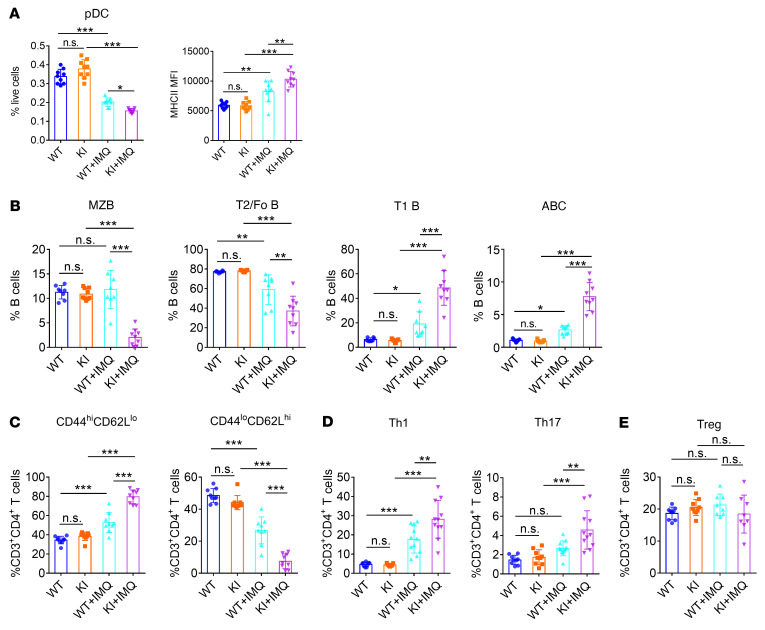
NCF1 p.R90H facilitates the overactivation of immune cells. Immune cell subsets in the spleen were analyzed by FACS. (**A)** Percentage and MHCII expression in pDCs (*n =* 9). (**B**) Proportion of B cell subsets (*n =* 9). (**C**) Proportion of activated splenic CD4^+^ T cell subsets (*n =* 9). (**D**) Proportion of splenic Th1 cell, Th17 cell, and (**E**) Treg subsets (*n =* 8–11). Error bars represent the SEM. *P < 0.05, **P < 0.01, and ***P < 0.001, by 1-way ANOVA with Tukey’s multiple-comparison test.

To further clarify the immunological changes as a result of NCF1 p.R90H, we evaluated the proportion and activation of pDCs and lymphocytes in the spleen. In IMQ-induced lupus model, the percentage of pDCs decreased, which was similar to the finding that the number of circulating pDCs decreased as a result of migration to the skin in patients with SLE (5). We detected a greater reduction in the percentage of pDCs and higher expression of MHCII in splenic pDCs of KI mice, indicating an overactivation of pDCs in KI lupus mice (Figure 4A and Supplemental Figure 5A).

It has been reported that lupus mice have abnormal B cell development, including loss of the marginal zone (MZ) B cell population and transitional 2 (T2)/follicular (Fo) B cells, together with an increase in transitional 1 (T1) B cells and an emergence of age-associated B cells (ABCs) (32–34). We found a similar change in WT and KI mice upon lupus induction (Figure 4B and Supplemental Figure 5, B and C). Additionally, the proportion of MZB and T2B cells further declined, whereas that of T1B cells and ABCs further increased in KI lupus mice compared with WT lupus mice. Meanwhile, CD44^hi^CD62L^lo^ activated CD4^+^ T cells increased at the expense of CD44^lo^CD62L^hi^ naive CD4^+^ T cells, which decreased (Figure 4C and Supplemental Figure 6A). Intracellular staining confirmed an elevation in the generation of Th1 and Th17 cells in KI lupus mice (Figure 4D and Supplemental Figure 6B). In contrast, there was no significant difference in the percentage of Tregs between WT and KI lupus mice (Figure 4E and Supplemental Figure 6C). Taken together, the immune cells of KI mice showed a more inflammatory state compared with those of WT mice.

To confirm whether pDCs were involved in the regulation of aggravated lupus symptoms in KI mice, we adopted a method similar to that used in a previous study by depleting pDCs in vivo using anti-PDCA1 antibodies (30). Mice were injected intraperitoneally with InVivoMAb anti-mouse antibody every 4 days throughout the 8 weeks of IMQ topical treatment. The InVivoMAb rat IgG2b isotype control was used as the control. The depletion efficiency of pDCs was confirmed by FACS (Supplemental Figure 7A). Consistent with previous reports, we found that clearance of pDCs greatly alleviated the progression of lupus, with reduced splenomegaly, resolved glomerulonephritis, and decreased autoantibodies (Supplemental Figure 7, B–D). The ISGs were obviously suppressed (Supplemental Figure 7E). The activation of ABCs and Th17 cells was also reduced after pDC depletion (Supplemental Figure 7F). More important, we noticed that there was no significant difference between WT and KI mice after pDC depletion in the severity of features, including spleen weights, glomerulonephritis, anti-dsDNA antibody levels, ISG expression, and the percentage of ABC and activated Th17 cell subsets. Taken together, depletion of pDCs eliminated the difference in lupus manifestation between WT and KI mice, supporting the critical role of pDCs in the pathogenesis of NCF1 variation.

### NCF1 levels are inversely related to the expression of IFN-I in human primary pDCs.

After confirming the function of NCF1 p.R90H in murine pDCs, we turned to the study of human pDCs. First, we were interested in determining the change in *NCF1* expression in IFN-I–producing pDCs. Single-cell transcriptome profiling (scRNA-Seq) has enabled high-resolution mapping of cellular heterogeneity, development, and activation states in diverse systems (35–37). Stochastic patterns of gene expression among cells within a homogeneous population were considered to be at the core of how the immune system can produce such a breadth of responses to maintain homeostasis and battle infections (35, 36). The transcriptional heterogeneity of a single DC subset in response to simultaneous stimulation provides us the opportunity to study the functional differences among multiple samples on a single-cell scale, where 1 cell serves as an independent sample.

**Figure 5:**
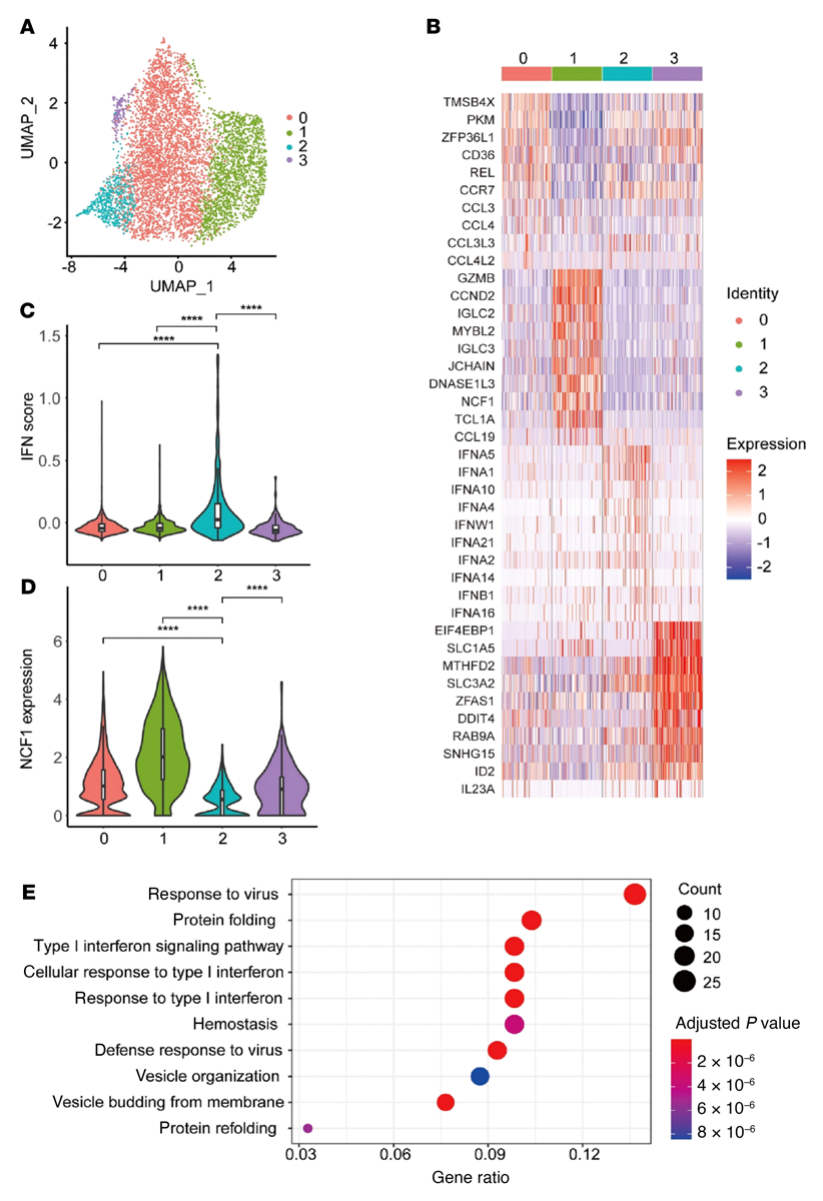
Single-cell analysis of pDCs. Human primary pDCs were stimulated with R848 for 12 hours and subjected to 10X Genomic detection. (**A**) UMAP plots of single cells. (**B**) Characteristic genes of the 4 pDC clusters. (**C**) Enrichment of IFN pathway genes. (**D**) Expression of *NCF1* in the 4 pDC clusters. (**E**) KEGG analysis of the featured genes in cluster 2. ****P < 0.0001, by Wilcoxon test.

To verify our hypothesis, we explored the correlation between *NCF1* expression and pDC function using scRNA-Seq. Human primary pDCs were sorted and challenged with R848 for 12 hours and then subjected to 10X Genomic sequencing (Supplemental Figure 8A). Four transcriptionally different clusters were obtained by unsupervised analysis (Figure 5A). The cluster 0 showed higher expression of cytokines and cytokine receptors, indicating that this group has a strong migratory and chemotactic ability (Figure 5B). Cluster 1 had characteristic expression of *GZMB* and *NCF1*. It was reported that GZMB^+^ pDCs exerted regulatory effects by inhibiting the expansion of T cells (38). Thus, this population may be “tolerant” pDCs with impaired antiviral ability. The higher *NCF1* levels in this group were also consistent with our findings that NCF1 is a negative regulator of pDC activation. Cluster 3 showed detectable *IL23* and *ID2*, which are normally expressed in myeloid DCs. IL-23 secreted by pDCs has been shown to be involved in the pathogenesis of IMQ-induced contact dermatitis (39). Of note, cluster 2 was specifically enriched by the expression of multiple IFN-I genes. So, cluster 2 represented the pDCs responsible for IFN-I production at this stage. The enrichment of IFN-I–related pathways and lowest expression of *NCF1* were confirmed in cluster 2 (Figure 5, C–E). Meanwhile, the expression levels of other NOX2 subunits, including *NCF4*, *CYBA*, and *RAC2*, were also lowest in cluster 2 (Supplemental Figure 8B). Therefore, the change in expression levels of *NCF1* in pDCs was opposite that of IFN-I–related genes, consistent with our previous report that NCF1 is an inhibitor of pDC activation (40). In line with this, a recent study that mapped the heterogeneity of PBMCs from patients with juvenile lupus erythematosus at the single-cell level reported that a specific pDC subcluster (179 of 655 cells) contributed to the IFN signature in lupus (41).

### The NCF1 p.R90H variant associates with the immunologic change seen in patients with SLE.

**Figure 6:**
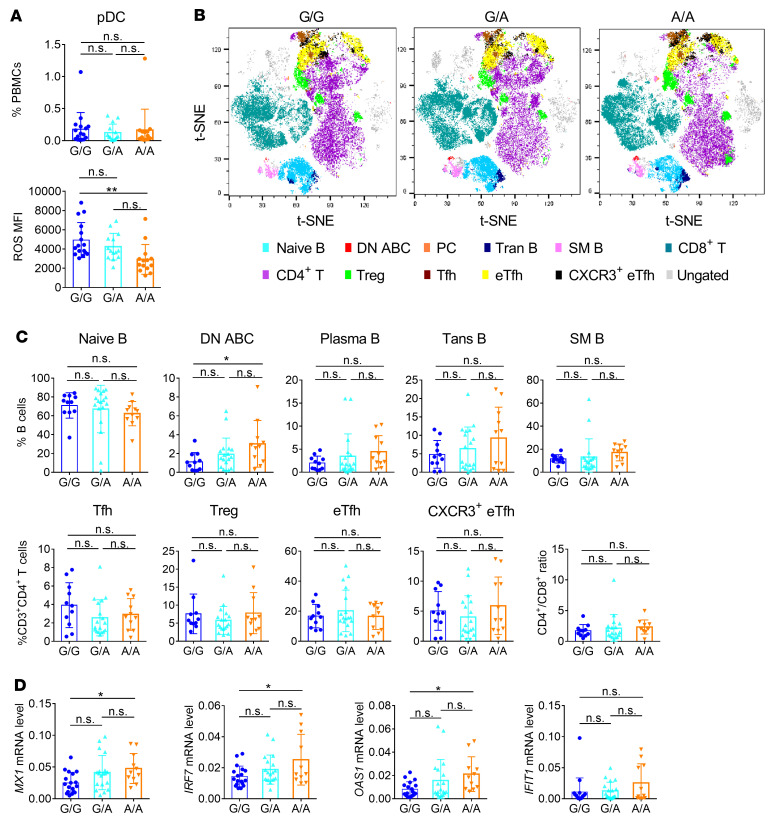
NCF1 p.R90H variant associates with the immunologic change in patients with SLE. (**A**) Percentage and ROS levels of pDCs in the peripheral blood of patients with SLE who carry the G/G (*n =* 16), G/A (*n =* 14), or A/A (*n =* 14) allele. (**B**) FACS analysis of B cell and T cell subsets in patients with SLE. Total B cells (CD19^+^); naive B cells (CD19^+^, CD27^–^, IgD^+^); DN ABCs (CD19^+^, CD24^–^, IgD^–^, CD27^–^, CD11c^+^); plasma cells (PCs) (CD19^+^, CD38^hi^, CD24^lo^); transitional B cells (Trans B) (CD19^+^, CD38^hi^, CD24^hi^); switched memory B cells (SM B) (CD19^+^, CD27^+^, IgD^–^); total T cells (CD3^+^); CD4^+^ T cells (CD3^+^, CD4^+^); CD8^+^ T cells (CD3^+^, CD8^+^); Tfh cells (CD3^+^, CD4^+^, CD127^+^,CD45RA^–^, CD25^–^, CXCR5^+^, PD1^hi^); Tregs (CD3^+^, CD4^+^, CD25^+^, CD127^–^); eTfh cells (CD3^+^, CD4^+^, CXCR5^–^, PD1^+^); and CXCR3^+^ eTfh cells (CD3^+^, CD4^+^, CXCR5^–^, PD1^+^, CXCR3^+^). Shown are *t*-distributed stochastic neighbor embedding (*t*-SNE) maps of 3 groups with 3 representative patients per group. (**C**) Statistics of B and T cell subsets, and the ratio of CD4^+^ T cells to CD8^+^ T cells are shown. (G/G, *n =* 11; G/A, *n =* 18; A/A, *n =* 11). (**D**) MRNA levels of *Mx1*, *Irf7*, *Oas1*, and *Ifit1* in SLE PBMCs (G/G, *n =* 18; G/A, *n =* 19; A/A, *n =* 11). The expression of mRNAs was normalized to the housekeeping gene *RPL13a* by calculating 2^–ΔCt^. Error bars represent the SEM. *P < 0.05 and **P < 0.01, by 1-way ANOVA with Tukey’s multiple-comparison test.

We have demonstrated that NCF1 p.R90H is a mutation with impaired function and that the reduced expression of NCF1 was closely related with pDC overactivation. Thus, we next verified the association of the NCF1 p.R90H variant with the immunologic change in patients with SLE by examining the composition of key immune cells in patients with G/G, G/A, or A/A alleles. We observed no significant difference in the proportion of pDCs among these patients (Figure 6A). Instead, ROS levels in A/A pDCs were statistically lower than those in G/G and G/A pDCs (Figure 6A). B cell analysis revealed that double-negative (DN) ABCs increased in A/A patients (Figure 6, B and C, and Supplemental Figure 9). The proportions of other B cell subsets, such as transitional B cells and memory B cells, remained unchanged (Figure 6, B and C). Considering that pDCs tend to augment the maturation of B cells via TLR7- and TLR9-driven IFN-I production (42), the elevation of autoreactive B cell generation in A/A patients could be attributed to the overactivation of pDCs. Besides, the percentages of T cell subsets, including follicular T helper (Tfh) cells, Tregs, extrafollicular T helper (eTfh) cells, CXCR3^+^ eTfh cells, and CD8^+^ T cells, were found to be similar among the 3 genotypes (Figure 6, B and C). Moreover, we detected the expression of ISGs in PBMCs. We found that mRNA levels of *MX1*, *IRF7*, and *OAS1* were higher in A/A PBMCs (Figure 6D). As IRF7 is a critical transcriptional factor enriched in pDCs, the enhanced *IFN-I* signature and *IRF7* expression indicated greater activation of pDCs in A/A patients. Notably, there was no significant difference between G/A and G/G patients with respect to the proportion of pathogenic lymphocytes and the expression of ISGs, supporting the idea that G/A heterozygous pDCs were functionally normal (Figure 6, A–D).

### Pharmaceutical application of HCQ alleviates the aggravation of NCF1 p.R90H in the lupus model.

Since the above results verified that NCF1 p.R90H was associated with SLE and detrimental for lupus, we believe it is of great importance to apply strategies to prevent the pathogenesis of the variant. By searching for drugs on the market and at the clinical research stage, we noticed that HCQ was a common drug capable of alkalizing the endosome (43, 44). As mentioned previously, NCF1 p.R90H facilitated pDC activation through the regulation of endosomal acidification. Thus, we decided to test the effect of HCQ on KI pDCs. Addition of HCQ suppressed the expression of IFN-α, IFN-β, TNF-α, and IL-6 in both G/G and A/A pDCs (Supplemental Figure 10, A and B). More important, HCQ treatment eliminated the difference in the activation of pDCs between 2 genotypes at the concentration of 1 μM.

**Figure 7:**
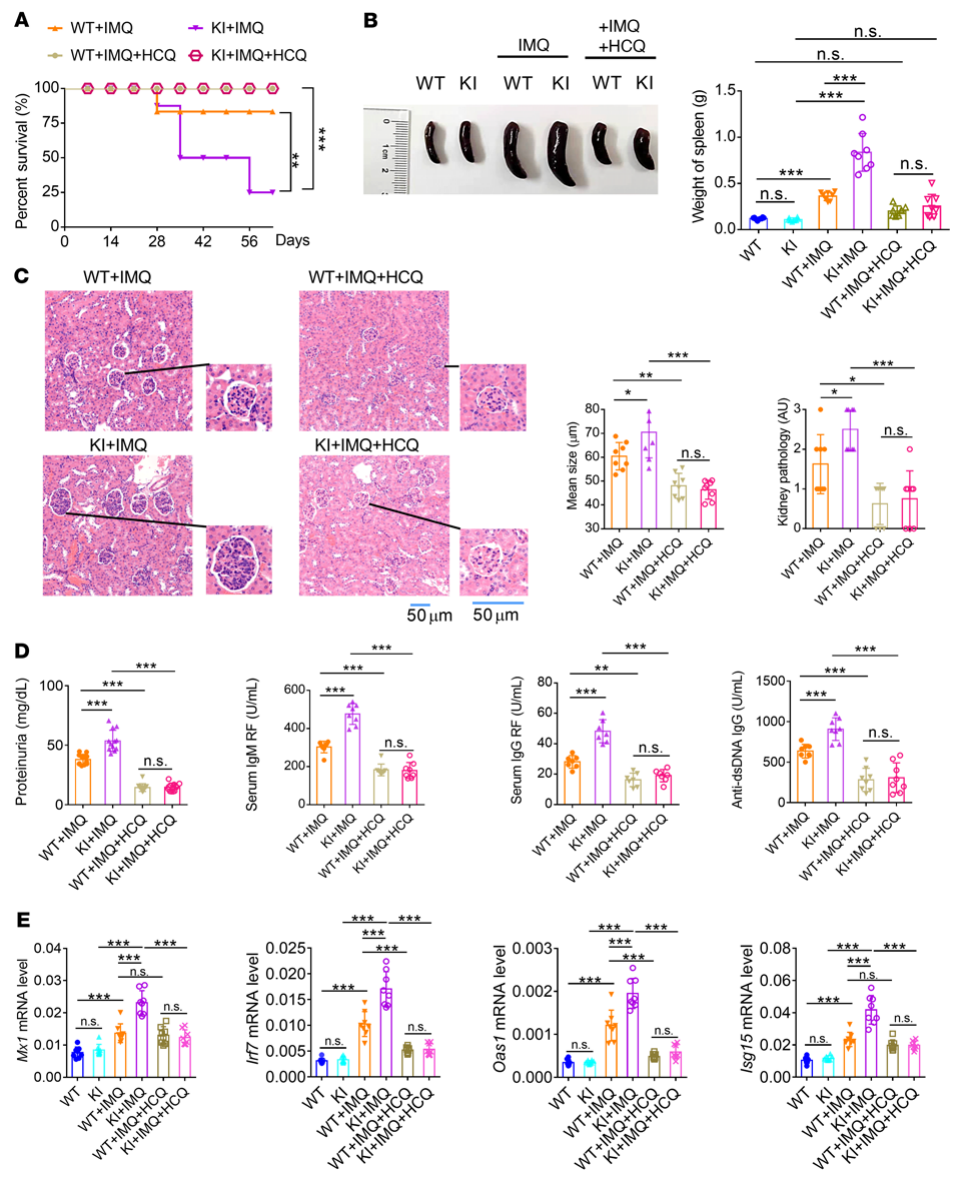
Pharmaceutical application of HCQ alleviates the aggravation of NCF1 p.R90H in a lupus model. WT and KI mice were treated with IMQ for 7 weeks. (**A**) Survival of WT lupus mice (WT+IMQ), KI lupus mice (KI+IMQ), HCQ-treated WT lupus mice (WT+IMQ+HCQ), and HCQ-treated KI lupus mice (KI+IMQ+HCQ). *n* = 20 mice per group. (**B**) Image and weights of spleens (*n =* 8). (**C**) H&E staining, glomerulus size, and pathological scores for kidneys (*n =* 8). Scale bar: 50 μm. (**D**) Levels of proteinuria (*n =* 12), serum RF IgM and IgG, and anti-dsDNA IgG (*n =* 8). (**E**) MRNA levels of *Mx1*, *Irf7*, *Oas1*, and *Isg15* in renal tissues (*n =* 8). The expression of mRNAs was normalized to the housekeeping gene *Actb* mRNA by calculating 2^–ΔCt^. Error bars represent the SEM. *P < 0.05, **P < 0.01, and ***P < 0.001, by log-rank test (**A**) or 1-way ANOVA with Tukey’s multiple-comparison test (**B**–**E**).

To further study the therapeutic effect of HCQ in vivo, HCQ was orally delivered into WT and KI lupus mice. Application of HCQ significantly improved the survival of KI lupus mice (Figure 7A), accompanied by the remission of splenomegaly (Figure 7B). Nephritis was obviously reduced in the HCQ treated groups as well, with less infiltration of lymphocytes and smaller glomeruli (Figure 7C). Furthermore, there was no difference in the renal pathology between WT and KI lupus mice following the HCQ treatment (Figure 7C). Consistent with this, the proteinuria and autoantibodies in the WT and KI lupus mice fed HCQ were reduced to similarly low levels (Figure 7D). Moreover, HCQ suppressed the induction of ISGs in vivo (Figure 7E). Thus, HCQ proved to be an effective drug for alleviating the lupus symptoms aggravated by NCF1 p.R90H.

**Figure 8:**
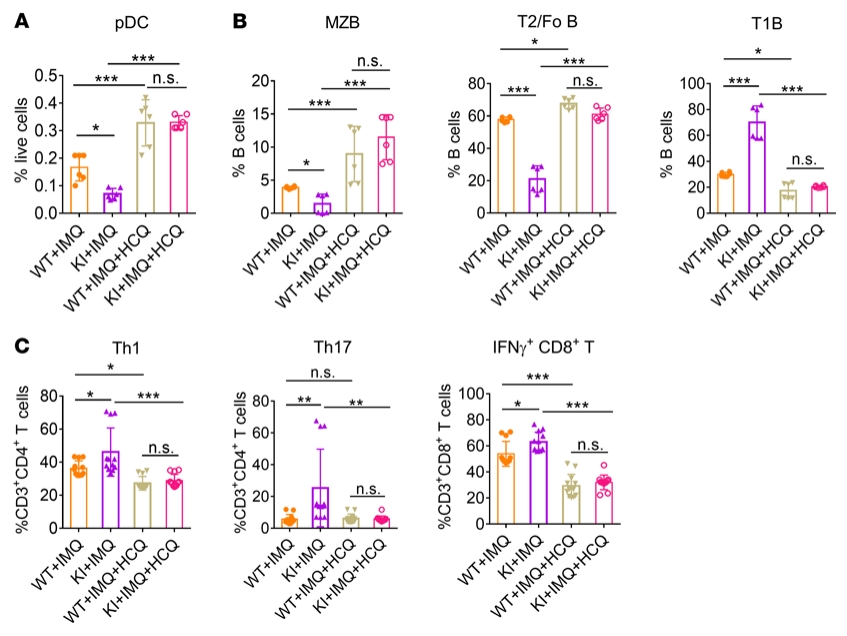
HCQ alleviates the overactivation of immune cells in vivo. The activation of immune cells from mice in Figure 7 was analyzed by FACS. Percentage of (**A**) splenic pDCs, (**B**) B cell subsets, and (**C**) T cell subsets (*n =* 6). *P < 0.05, **P < 0.01, and ***P < 0.001, by 1-way ANOVA with Tukey’s multiple-comparison test. Error bars represent the SEM.

We also monitored changes in the activation of immune cells. HCQ treatment suppressed the inflammatory response, including the recovery of pDCs in the spleen, increased the proportion of MZB and T2B cells, and decreased the proportion of T1B cells (Figure 8, A and B, and Supplemental Figure 11, A and B). The generation of pathogenic T cell subsets, such as Th1, Th17, and IFN-γ^+^CD8^+^ T cells, was hampered upon HCQ treatment (Figure 8C and Supplemental Figure 11C). The proportion of ABCs was also found to be reduced by HCQ in vivo (Supplemental Figure 12, A and B). More important, we observed no significant difference in the composition of splenic pDCs, inflammatory B cell subsets, or T cell subsets between WT and KI lupus mice upon HCQ application, confirming the special role of HCQ in alleviating the excessive self-responses resulting from the NCF1 p.R90H mutation.

In addition, although NCF1 has been proven to be critical for the proinflammatory function of neutrophils, no significant change in the percentage of neutrophils was observed between WT and KI lupus mice, with or without HCQ treatment (Supplemental Figure 12, C and D). In contrast, the percentage of monocytes declined in KI lupus mice compared with WT lupus control mice, and HCQ further hampered the recruitment of monocytes (Supplemental Figure 12, C and D).

### HCQ eliminates the functional promotion of NCF1 p.R90H in human samples.

As HCQ has been proven to be an effective drug in the NCF1 p.R90H–aggravated lupus mouse model, we proceed to investigate the effect of HCQ on in vitro PBMCs from patients with different genotypes. PBMCs were isolated from individuals from 4 independent families harboring G/A and A/A alleles (Supplemental Figure 13A). These samples were then subjected to CpG stimulation with or without HCQ treatment. Compared with G/A PBMCs, the A/A PBMCs manifested an increased tendency to produce IFN-α, although there was no statistical difference, probably because of the limited sample size (Supplemental Figure 13B). Furthermore, application of HCQ inhibited IFN-α secretion. More important, the difference in IFN-α expression levels between the 2 groups was eliminated.

Therefore, the homozygous NCF1 p.R90H variant closely correlated with autoimmune responses in patients with SLE, and HCQ may serve as a potential drug for the treatment of NCF1 p.R90H–aggravated lupus (Supplemental Figure 14).

## Discussion

In this study, we investigated the function of *NCF1* SNP rs201802880 in the progression of lupus. We found that the NCF1 p.R90H variant facilitated endosomal cleavage and activation of TLR7 and TLR9 in the endosome, thereby boosting the TLR pathway and IFN-I production, which resulted in more serious autoimmune responses and aggravated lupus symptoms.

Using a mouse carrying a natural mutation in the *Ncf1* gene (*Ncf1^m1J^*), the importance of NCF1 has been revealed for various autoimmune diseases, including arthritis, experimental autoimmune encephalomyelitis (EAE), SLE, psoriasis, etc. (17). NCF1, as part of the NOX2 complex, prevents excessive T cell responses through its generation of ROS (45, 46). In neutrophils, an NCF1-associated oxidative burst was reported to facilitate the formation of NETs. NETs were generally considered to be pathogenic autoantigens in autoimmune disease (47). Besides, NCF1 could suppress activation of the JAK/STAT pathway and inhibit the production of proinflammatory cytokines by hampering the ubiquitylation of nuclear factor erythroid 2 p45–related factor 2 (NRF2) (48). Our study emphasizes its critical role in the regulation of pDC function. Generation of ROS by NOX2 and their subsequent dismutation into hydrogen peroxide consumes protons, resulting in increased pH. Upon TLR stimulation, NCF1 is activated and then translocated to the endosome. By elevating endosomal pH, NCF1 shuts off TLR recognition and protects pDCs from persistent overactivation. The NCF1 p.R90H mutation interrupts the negative regulation and leads to autoimmunity. Moreover, ROS has been verified to be a powerful inhibitor of pDC activation, and even fairly low concentration of H_2_O_2_ is able to significantly suppress the TLR-driven production of IFN-I (49, 50).

We demonstrated that the NCF1 p.R90H variant augments the activation of pDCs. Since there were no pDC-specific *CRE* mice, and it seemed impractical to construct cell-specific SNP mice, it would be a challenge to determine the role of NCF1 p.R90H specifically in pDCs using a lupus model. As the effects of NCF1 have been confirmed in both lymphoid and myeloid cells, we could not conclude that the aggravation of lupus by NCF1 p.R90H was exclusively dependent on its function in pDCs. However, our results suggested that overactivated pDCs played a central role in the NCF1 p.R90H promotion of SLE. First, the IMQ-induced lupus model used in our study was pDC dependent, and antibody-mediated clearance of pDCs in vivo hampered the occurrence of lupus (30). Depletion of pDCs greatly eliminated the difference in lupus progression between WT and KI mice. Second, although NCF1 has been reported to regulate the function of neutrophils and Tregs, we observed no significant change in the proportion of these cells between WT and KI lupus mice. Greater reductions in pDC percentages and excessive activation of T and B cells were confirmed in KI lupus mice. pDCs facilitate autoimmune disease by boosting Th1 generation and B cell maturation. Considering the higher expression of ISGs in KI renal tissues, which is related to pDCs and not lymphocytes, the elevation in the proportion of Th1 cells and ABCs was more likely the result of pDC dysfunction. Third, NCF1 p.R90H was associated with increased ABCs, indicating excessive B cell activation in patients with SLE who carry a homozygous risk allele. In contrast, there was no obvious change in T cell subsets. Given that the expression of IFN-I–induced genes was higher in A/A patients, we propose that pDCs are critical for NCF1 p.R90H–driven lupus aggravation in humans. Further studies of the reconstruction of pDC function, by transferring SNP pDCs into pDC-deficient (e.g., *TCF4^–/–^*) mice, for instance, will help us to reach more solid conclusions (51). Interestingly, during the revision of this article, a study was published reporting that NCF1 p.R90H promoted lupus through an enhanced Tfh2 response induced by a defective efferocytosis of macrophages when a pristane-induced lupus model was used (52). Different models will help clarify the multiple roles of NCF1 in lupus.

In addition to IFN-I, type III IFNλs were also found to be involved in the pathogenesis of an IMQ-induced lupus models (31). By activating the production of chemokines by keratinocytes and mesenchymal cells, IFNλ was found to play a tissue-specific pathological role in lupus. In addition to type I IFNs, pDCs can also produce type III IFNs/IFNλs in response to TLR7/-8 and TLR9 stimulation. Based on our RNA-Seq data, we found that *IFNλ2* and *IFNλ3* expression was increased in A/A pDCs.

In one study, pDCs from patients with SLE were divided into 2 subgroups by extensive RNA-Seq based on the relative expression of IFN-inducible genes (53). The IFN^hi^ pDC population showed enriched expression of IFN-related genes that could be mapped to our cluster 2 population. The reported IFN^lo^ subpopulation was enriched for genes involving IL-10 signaling, cell migration, and pathogen interaction pathways, covering features of our clusters 0, 1, and 3. More important, the researchers found that genes related to cellular oxidative stress were commonly enriched in IFN^hi^ and IFN^lo^ pDCs from patients with SLE and that ROS significantly inhibited the activation of pDCs. Their extensive studies suggest the existence of an “exhausted transcriptional phenotype” in SLE pDCs that is caused by higher ROS levels. Thus, the potential resistance to “pDC exhaustion” in patients with SLE who have the A/A genotype may exacerbate disease progression. Moreover, based on the expression of CD8α and CD8β, murine pDCs were separated into 3 stable subsets (54). The CD8α^+^CD8β^+^ and CD8α^+^CD8β^–^ subsets were tolerogenic, whereas the CD8α^–^CD8β^–^ subset was immunogenic. We isolated the CD8α^–^ and CD8α^+^ pDCs from spleens and detected the endosomal localization of NCF1 (Supplemental Figure 15). The results showed no significant difference between these pDC subsets.

SLE is an autoimmune disease with genetic susceptibility. Studies of genetic polymorphisms have identified various genetic variants associated with SLE, but these findings were rarely transferred to the clinical management of SLE. As one of the principal medications for SLE treatment, HCQ exerts its therapeutic effects through the alkalinization of intracellular lysosomes, thus limiting antigen-presenting cell function (44, 55). With this in mind, we have connected the genetic polymorphism with the mechanism of action of HCQ in the current study. First, compared with other well-known susceptibility loci located in *IRF5*, *TLR7*, *IRF7*, etc., *NCF1* SNP rs201802880 is at the top of genetic variants strongly associated with SLE. Second, NCF1 p.R90H has been found to be closely related with a variety of autoimmune diseases including SLE, RA, SS, and others (15). Correspondingly, HCQ has been verified to be effective in these diseases. Third, NCF1 p.R90H aggravates lupus by acidifying the pH of endosomes/lysosomes and facilitating the persistent activation of TLR signaling. HCQ is an alkalinizing drug that accumulates in late endosomes/lysosomes and antagonizes excessive the inflammatory response by increasing the endosomal/lysosomal pH. Although not proved in the current study, we would imagine using the *NCF1* SNP rs201802880 as a genetic marker for patient stratification in studies focusing on HCQ efficacy in SLE. Moreover, determining whether the varying prevalence of the SNP in different ethnic groups can partially explain the ethnicity differences in flare risks after HCQ withdrawal will require further investigation (15, 56). Future clinical studies are needed to validate the role of the *NCF1* SNP rs201802880 in stratifying patients with SLE when evaluating the benefit of HCQ.

In addition to *NCF1*, other hypomorphic NOX2 subunits associated with lupus might also contribute to the ROS change in pDCs, of which SNPs in *NCF2* were reported by different groups (57–59). Furthermore, haploinsufficiency of *Ncf2* has been demonstrated to be sufficient to accelerate the progression of lupus (60). Although the major *NCF2* variant rs17849502 was not found in our patients’ samples, it is possible that other variants of NOX2 subunits in different populations might also contribute to the reduced ROS in pDCs that leads to aggravated lupus through a mechanism similar to that identified in this study. More research in mice harboring single or multiple SNPs will help clarify the effect of other hypomorphic forms of NOX2 on SLE.

## Methods

### Patients.

A total of 49 patients with SLE were recruited from Renji Hospital of the Shanghai Jiao Tong University School of Medicine. All patients fulfilled the 1997 American College of Rheumatology (ACR) classification criteria for SLE. The SLE Disease Activity Index 2000 (SLEDAI-2k) and the renal SLEDAI (RSLEDAI) score (the total score of the 4 kidney-related parameters in SLEDAI-2k) were calculated on the basis of chart reviews. Patient information is provided in Supplemental Table 1, and patient information for the SLE families is shown in Supplemental Table 2. Human primary pDCs for scRNA-Seq were obtained from a healthy 29-year-old woman (control).

### Mice.

*Ncf1*-KI mice were generated using the CRISPR/CAS9 technique. The sgRNA sequence was 5′-ACGAGCCGCTGAGAGTCGCC-3′, and the donor sequence was 5′-CATGGGTCTCTGGCTCCCCCACCCAGCACCCAGGTGGTTTGATGGGCAACGAGCAGCTGAGTCCCACCAGGGCACTCTCACTGAATACTTCAACGGCCTCATGGGACTGCCCGTGAAGAT-3′. *Cas9* mRNA, sgRNA, and donor DNA were coinjected into zygotes. Thereafter, the zygotes were transferred into the oviduct of pseudopregnant ICR (strain no. N000294, GemPharmatech) females at 0.5 dpc. Approximately 19–21 days after transplantation, all offspring of the ICR females (F0 mice) were identified by sequencing of tail DNA. Positive F0 mice were crossed with C57BL/6J mice to generate heterozygous KI mice. In addition, female mice at 10–12 weeks of age were used to carry out all experiments. All mice were bred and housed under specific pathogen–free conditions.

### IMQ-induced lupus model, HCQ treatment, and pDC depletion.

The lupus model was induced according to the previous reports with minor modifications (30). Briefly, the skin on the right ears of the mice was treated topically every other day, with 2 mg of 5% IMQ cream (Aldara, 3M Pharmaceuticals) for 6–12 consecutive weeks. Control mice were given the same dose of vehicle cream. Mice were treated with 10 mg/kg per day HCQ (Selleck Chemicals) by oral gavage during the induction period. For pDC depletion in vivo, mice were first injected intraperitoneally with 500 μg pure InVivoMAb anti-mouse antibody (Bio X Cell, BE0311) 4 days before IMQ application and then treated with antibodies every 4 days over the 8 weeks of topical treatment with IMQ. InVivoMAb rat IgG2b isotype control (Bio X Cell, BE0090) was used as the control.

### Cell culturing and stimulation.

Mouse primary pDCs from spleens were isolated by FACS as CD11c^int^SiglecH^+^PDCA1^+^ cells (FACSAria III, BD) and cultured in RPMI-1640 (Gibco, Thermo Fisher Scientific) containing l-glutamine supplemented with 10% FBS (Gibco, Thermo Fisher Scientific); 1% penicillin-streptomycin (Gibco, Thermo Fisher Scientific); and 0.1% 2-mercaptoethanol (Gibco, Thermo Fisher Scientific). For Western blotting, FLT3L-induced pDCs were used. Murine BM cells were cultured with 200 ng/mL FLT3L (Peprotech) for 9 days, and pDCs were sorted as CD11c^int^SiglecH^+^. Human primary pDCs were first enriched from PMBCs using the Human Diamond Plasmacytoid Dendritic Cell Isolation Kit II (Miltenyi Biotec) and then sorted by FACS. Cells were treated with 10 μg/mL R848 or 0.5 μM ODN2216 (InvivoGen), with or without the indicated inhibitors. The inhibitor concentrations were as follows: 2-DG, 1 μM; metformin, 1 μM; etomoxir, 200 μM; NAC, 1 mM; HCQ, 0.5 μM or 1 μM (Selleck Chemicals); BafA, 100 nM; and 2-GBI, 100 μM.

### Histology.

Mouse kidneys were fixed in formalin and embedded in paraffin, and tissue sections were stained with H&E. Pathology was assessed using Case Viewer software (3DHISTECH Ltd.) and scored in a blinded manner on a scale of 0–3 for increasing severity of glomerulonephritis as follows: (a) minimal damage to the glomerular basement membrane (GBM) and little to no cellular infiltrates; (b) intermediate degree of cellular infiltration with moderate damage to the GBM; (c) extensive cellular infiltration and severe destruction of the GBM, which may include the presence of casts indicating proteinuria.

### Measurement of cytokines, proteinuria, and autoantibodies.

Murine IFN-α and IFN-β levels were detected using the Mouse IFN-α ELISA kit and the Mouse IFN-β ELISA kit (both from R&D Systems), respectively. Murine TNF-α and IL-6 were quantified using the ELISA MAX Deluxe Set Mouse IL-6 and ELISA MAX Deluxe Set Mouse TNF-α kits (BioLegend). Human IFN-α was determined with the Human IFN-α Multi-Subtype ELISA Kit (R&D Systems). Proteinuria was examined using the LBIS Mouse Urinary Albumin Assay Kit (Shibayagi). RF-IgM, RF-IgG, and anti-dsDNA antibody levels in serum were measured by ELISA (LBIS Mouse RF-IgM, RF-IgG, and anti-dsDNA antibody kits, Shibayagi). All detection was performed according to the manufacturer’s instructions.

### Flow cytometric analysis and ROS detection.

For mice, single splenic cells were prepared by digesting the tissue fragments with collagenase IV (MilliporeSigma) and DNase I (MilliporeSigma) and subjected to FACS analysis by Fortessa (BD Biosciences). Human PBMCs were isolated with Ficoll-Paque PLUS (1.077 g/cm^3^, GE Healthcare) through gradient centrifugation and analyzed by FACS. The ROS levels were detected with the OxyBURST Green H2DCFDA kit (Thermo Fisher Scientific) according to the protocol. Mitochondrial ROS levels were detected with MitoSOX Red (Thermo Fisher Scientific) according to the manufacturer’s instructions. The antibodies and reagents used are listed in Supplemental Table 3. Data were analyzed with FlowJo software.

### RNA-Seq.

Total RNA was extracted by TRIzol Reagent (Invitrogen, Thermo Fisher Scientific). Next, RNA libraries were prepared using the VAHTS mRNA-Seq, version 2, Library Prep Kit for Illumina (Vazyme) and sequenced in a HiSeqx 10 system (Illumina) on a 150 bp paired-end run. Genes with a statistically significant *P* value (< 0.05) by analysis of DEseq2 and a log_2_ fold change of 1 or greater or –1 or less were selected for further analysis. The Kyoto Encyclopedia of Genes and Genomes (KEGG) was used to determine the significant pathway of the selected genes. The RNA profiling data generated in this study are available in the NCBI’s Gene Expression Omnibus (GEO) database (GEO GSE176349).

### Single-cell sequencing.

Human primary PBMCs were obtained from a healthy 29-year-old woman. After sorting by FACS, approximately 12,000 pDCs were counted, and their viability was confirmed to be greater than 90%. Single-cell suspensions were loaded onto a 10X Genomics Chromium Single-Cell 3′ v2 chip to prepare the libraries according to the manufacturer’s protocol. The library quality was verified with an Agilent 2100 Bioanalyzer and Qubit fluorometric quantitation. Samples were sequenced on an Illumina NovaSeq 6000, with a sequencing depth of at least 50,000 reads per cell.

The raw fastq data were processed with CellRanger (version 2.1.1) using GRCh38 annotation (version 1.2.0) (61). Output from CellRanger was loaded into R, and Seurat (version 3.1.1) was used for further analysis (62). Cells with fewer than 1000 or more than 4500 expressed genes and a high percentage of mitochondrial genes (>5%) were removed. Peter Karchenko – the Pagoda way was also used to define outliers. Among them, 6010 cells passed the filtering process. After normalizing and scaling, we used “Find Variable Features” to identify 1000 highly variable genes. Then we used 992 highly variable genes (removing mitochondrial and ribosomal genes) for principal component analysis (PCA). Using “Elbow plot,” we selected the top 20 principal components for clustering and uniform manifold approximation and projection (UMAP) visualization with default parameters. For clustering, we set the resolution parameter at 0.2, and 4 clusters were shown. To obtain cluster-specific gene signatures, we identified differential expression analysis of each cluster with default parameters. IFN scores based on IFN gene sets were calculated for each cell as the average of scaled expression of these genes and corrected by subtracting the average of a large set of similar genes, as proposed by Tirosh et al. (63). The single-cell sequencing data are available in the GEO database (GEO GSE176392).

### Quantitative real-time RT–PCR.

cDNAs were prepared using reverse transcription (PrimeScript RT Reagent Kit, Takara) and amplified by real-time quantitative PCR with the primers shown in Supplemental Table 4. Amplification was conducted in an ABI PRISM 7900 Real Time PCR System (Applied Biosystems). The expression of murine and human mRNAs was respectively normalized to *Actb* and *RPL13a* mRNA by calculating 2^–ΔCt^, where Ct was the cycle threshold.

### NCF1-specific PCR and TaqMan genotyping.

*NCF1* genotyping was performed as described in our previous reports (15). Briefly, we first amplified an *NCF1*-specific fragment by targeting the GTGT sequence in exon 2 of *NCF1*. A PCR fragment diluted 1:10,000 was used as the template in a TaqMan assay for SNP genotyping. TaqMan assays were run on either ViiA 7 or the QuantStudio 7 Flex RT-PCR System (Thermo Fisher Scientific). Primer sequences are listed in Supplemental Table 4.

### Immunoblots.

pDCs (5 × 10^6^) were lysed using RIPA protein extraction buffer (Thermo Fisher Scientific). Cell lysates (10 μg/lane) were separated by 10% SDS-PAGE and transferred onto an Immobilon-P polyvinylidene difluoride membrane (MilliporeSigma). Then, the membrane was blocked with SuperBlock TBS blocking buffer (Thermo Fisher Scientific) and incubated with the antibodies listed in Supplemental Table 3. Gels were analyzed using ImageJ (NIH).

### Protein and phospholipid binding assay.

GST-tag NCF1 90R and GST-tag NCF1 90H were synthesized and purified by GenScript. Binding assays were performed as described previously (28). Briefly, 1-palmitoyl-2-oleoyl-sn-glycero-3-PC (POPC) (Cayman Chemical); 1-palmitoyl-3-oleoyl-sn-glycero-2-PE (POPE) (Cayman Chemical); and PtdIns(3,4)P2 (Cayman Chemical) (47.5:47.5:5) were mixed in chloroform. Phospholipids were dried using nitrogen and resuspended in 500 mL binding buffer (20 mM Tris, pH 7.4, 100 mM NaCl, 1 mM DTT). Protein and phospholipids were mixed in 100 mL reactions to yield solutions containing 1 mM total lipid and 5 mM protein. After incubation for 60 minutes at 20°C, the solution went through centrifugation for 15 minutes at 16,000*g* at room temperature. Supernatants were carefully removed, and the pellets were resuspended in SDS sample buffer. Both the pellets and the supernatants were analyzed by SDS-PAGE.

### Protein structure analysis.

The PX domain of NCF1 was obtained from the RCSB Protein Data Bank with the deposition ID 1KQ6 (https://www.rcsb.org/). The structures of NCF1 90R and 90H were analyzed using UCSF Chimera.

### Protein overexpression and knockdown.

For catalase overexpression, full-length cDNA of murine catalase was subcloned into the site between EcoRI and NotI enzymes of the pMYs-IRES-Puro plasmid (Cell Biolabs) and transduced into the platinum-E cell line to produce a retrovirus. Lineage^–^c-Kit^hi^ BM progenitors were infected with control or a catalase-overexpressing retrovirus and subjected to FLT3L induction for 8 days. Finally, pDCs were isolated and used for subsequent testing. For RAC2 knockdown, lentivirus expressing an shRNA specifically targeting murine *Rac2* (MilliporeSigma) was constructed and transduced into lineage^–^c-Kit^hi^ BM progenitors.

### Confocal fluorescence imaging.

Cells were fixed with 4% paraformaldehyde (PFA) for 15 minutes and treated for 10 minutes with PBS containing Triton X-100 (0.25%). The samples were then incubated with mouse IgG–blocking reagent (BioLegend), followed by the addition of goat anti-NCF1 antibody (1:50, Abcam) and rabbit anti-EEA1 antibody (1: 100, Abcam) for 1 hour and then washing. AF488 donkey anti–goat IgG antibodies (1:100) were then stained for 30 minutes and washed. Finally, AF647 goat anti–rabbit IgG antibodies (1:100) were incubated for 30 minutes at room temperature. To detect the colocalization of NCF1 and LAMP1, AF647 rat-mouse antibody (1:100, BioLegend) and a goat anti-NCF1 antibody (1:50, Abcam) were incubated for 1 hour and washed. Then, AF488 donkey anti–goat IgG antibody (1:100) was stained for 30 minutes at room temperature. After that, cells were incubated with DAPI (1:5000, BD Biosciences) for 5 minutes. Cells were then detached onto glass dishes and viewed and photographed on a confocal fluorescence microscope (THUNDER Imager Tissue and Leica TCS SP8, Leica Microsystems). All immunofluorescence was assessed in a blinded manner by 2 observers throughout the analysis process. The colocalization of NCF1 and EEA1 and of NCF1 and LMAP1 was calculated with ImageJ software (NIH).

### Endosomal pH detection.

PDCs were washed and incubated with prewarmed (37°C) lysosensor probe–containing medium (1 μM, LysoSensor Green DND-189, Thermo Fisher Scientific) for 1 hour. Then, the cells were washed and subjected to confocal analysis.

### Statistics.

For immunofluorescence assays, approximately 30–50 cells per treatment group from at least 3 independent experiments were analyzed to calculate the fluorescence in each cell. All data were analyzed using GraphPad Prism (GraphPad Software, version 6.0). A *P* value of less than 0.05 was considered significant. Data are presented as the mean ± SEM. Statistical analysis was performed with a 2-tailed, unpaired *t* test. Comparisons among multiple groups were performed using 1-way ANOVA with Tukey’s multiple-comparison test. The single-cell data were analyzed with the Wilcoxon test. Differences between survival curves were estimated by log-rank test.

### Study approval.

All human experiments were conducted in accordance with Declaration of Helsinki principles. Written informed consent was obtained from participants prior to inclusion in the study. Human studies were approved by the ethics committee of Renji Hospital of the Shanghai Jiao Tong University School of Medicine. All animal experiments were approved by the animal care committee of Renji Hospital.

## Author contributions

NS, HZ, and Z Ye designed the project. YM, JM, and CY performed the experiments, and HZ, YM, YH, and CY analyzed the data. Jun Li, CL, and GL established the mouse model. HD, Jia Li, and Z Yin collected the human samples. HZ, YM, CY, JM, LW, and NS prepared the manuscript.

## Supplementary Material

Supplemental data

## Version 1 07/05/2022

In-Press Preview

## Version 2 08/15/2022

Electronic publication
