# Supplementary material for: The NCF1 variant p.R90H aggravates autoimmunity by facilitating the activation of plasmacytoid dendritic cells
Source: J Clin Invest. 2022 Aug 15;132(16):e153619. doi: 10.1172/JCI153619 (PMC9374378; doi:10.1172/JCI153619)
Supplement: Supplemental data [file jci-132-153619-s061.pdf]

Supplemental Figures

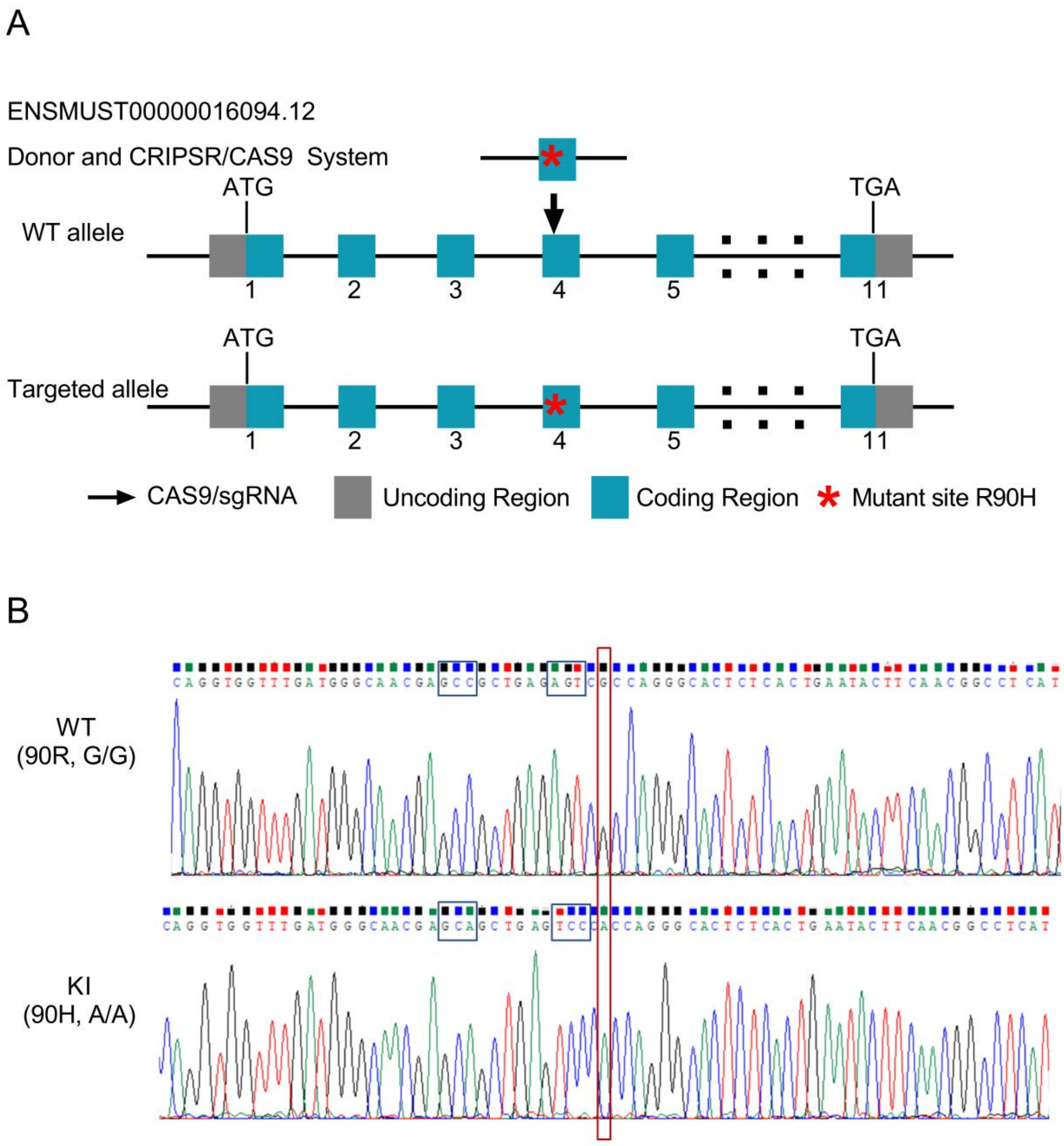

Supplemental Figure 1. Strategy of constructing KI mice. (A) KI mice were generated by using CRISPR/CAS9 gene editing and homologous recombination technique. (B) Verification of p.90R to p.90H mutation in KI mice. The black squares indicate the position of synonymous codon substitution.

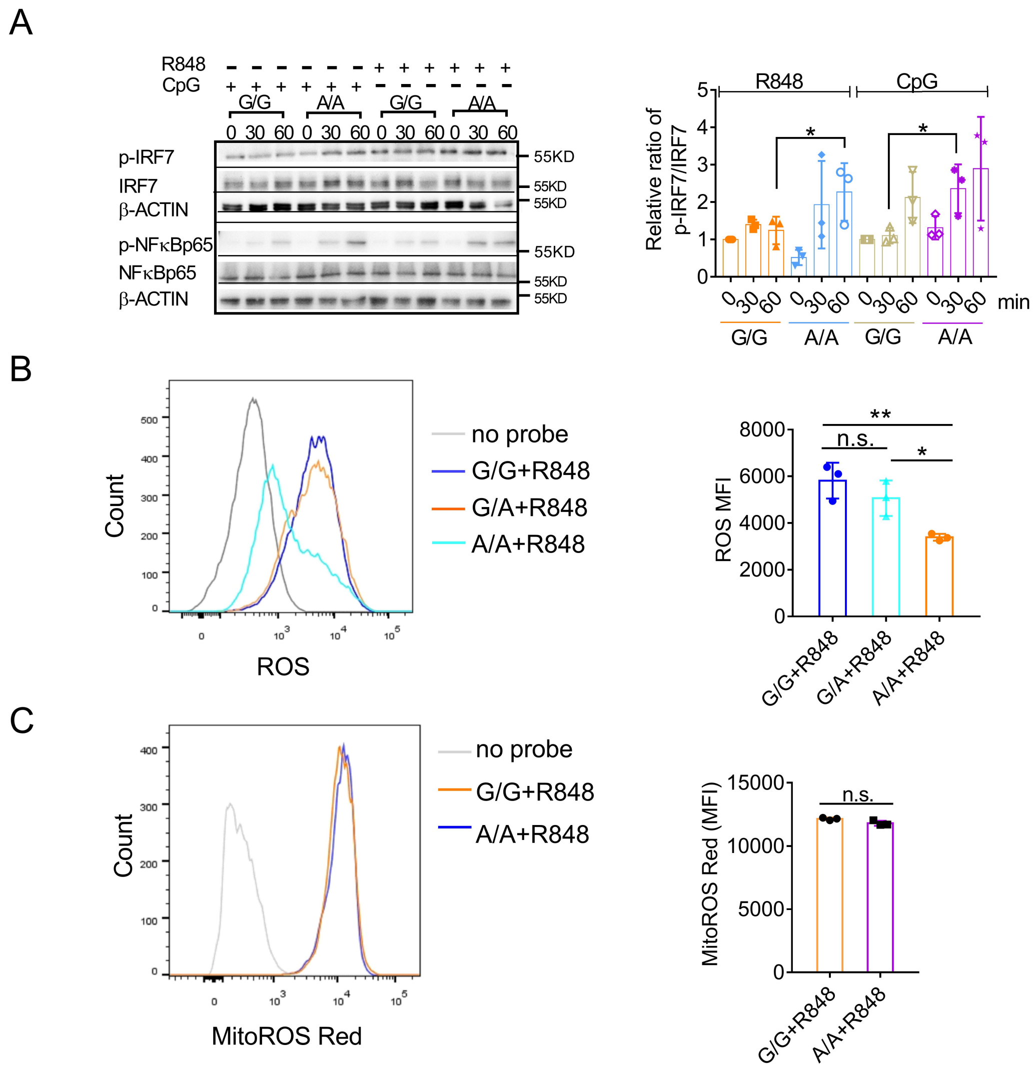

Supplemental Figure 2. The activation and ROS levels of pDCs with different alleles. (A) G/G and A/A pDCs were generated by FLT3L induction. Then, pDCs were sorted and stimulated with R848 or CpG for indicated time. The levels of phospho-IRF7 (p-IRF7), IRF7, phospho-NFκB p65 (p-NFκBp65) and NFκBp65 were detected. β-ACTIN was used as loading control. *p* value was determined by a two-tailed paired *t* test. Error bars represent SEM. (*p<0.05). n=3. (B, C) Splenic pDCs from G/G, G/A and A/A mice were stimulated with R848 for 1 hour and then analyzed by FACS. The levels of (B) ROS and (C) mitochondrial ROS are shown. *p* values were determined by one-way ANOVA with Tukey's multiple comparison test. Error bars represent SEM. (*p<0.05, **p<0.01, n.s. not significant).

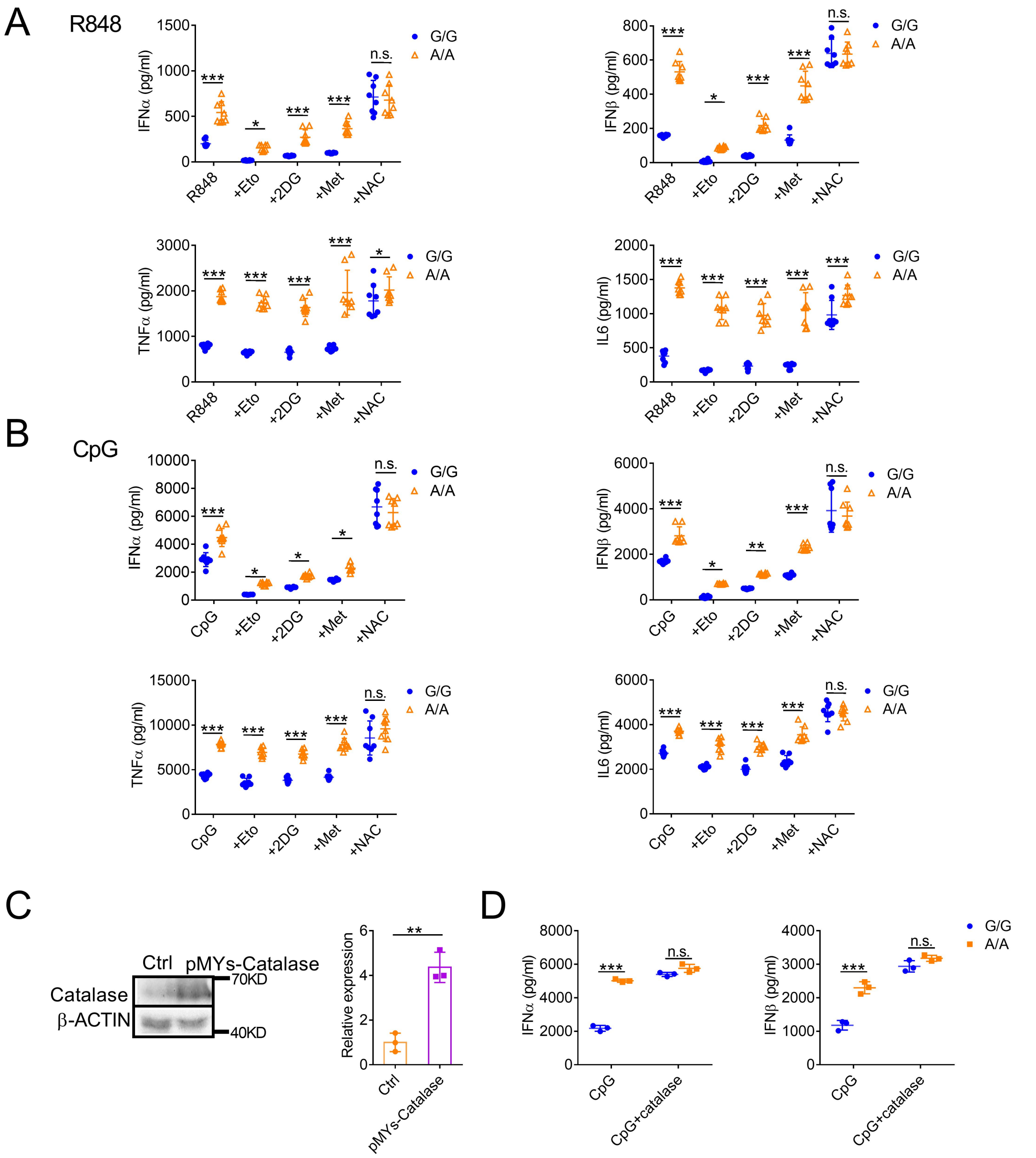

Supplemental Figure 3. ROS scavenger eliminates the difference in the production of IFN-I resulted from NCF1 p.R90H. PDCs were pretreated with or without etomoxir (Eto), 2-DG, metformin (Met), or NAC for 2 hours and then stimulated with (A) R848 or (B) CpG for 24 hours. The expression of IFNα, IFNβ, TNFα and IL6 were detected. n=8. (C, D) Bone marrow progenitors from WT or KI mice were transfected with empty or Catalase overexpressing lentivirus, and followed by 8-day FLT3L induction. PDCs were sorted and stimulated with CpG for 24 hours. (C) The overexpression of Catalase. *p* value was determined by a two-tailed paired *t* test. Error bars represent SEM. (*p<0.05). n=3. (D) The expression of IFNα and IFNβ. n=3. *p* values were determined by two-way ANOVA. Error bars represent SEM. (*p<0.05, **p<0.01, n.s. not significant).

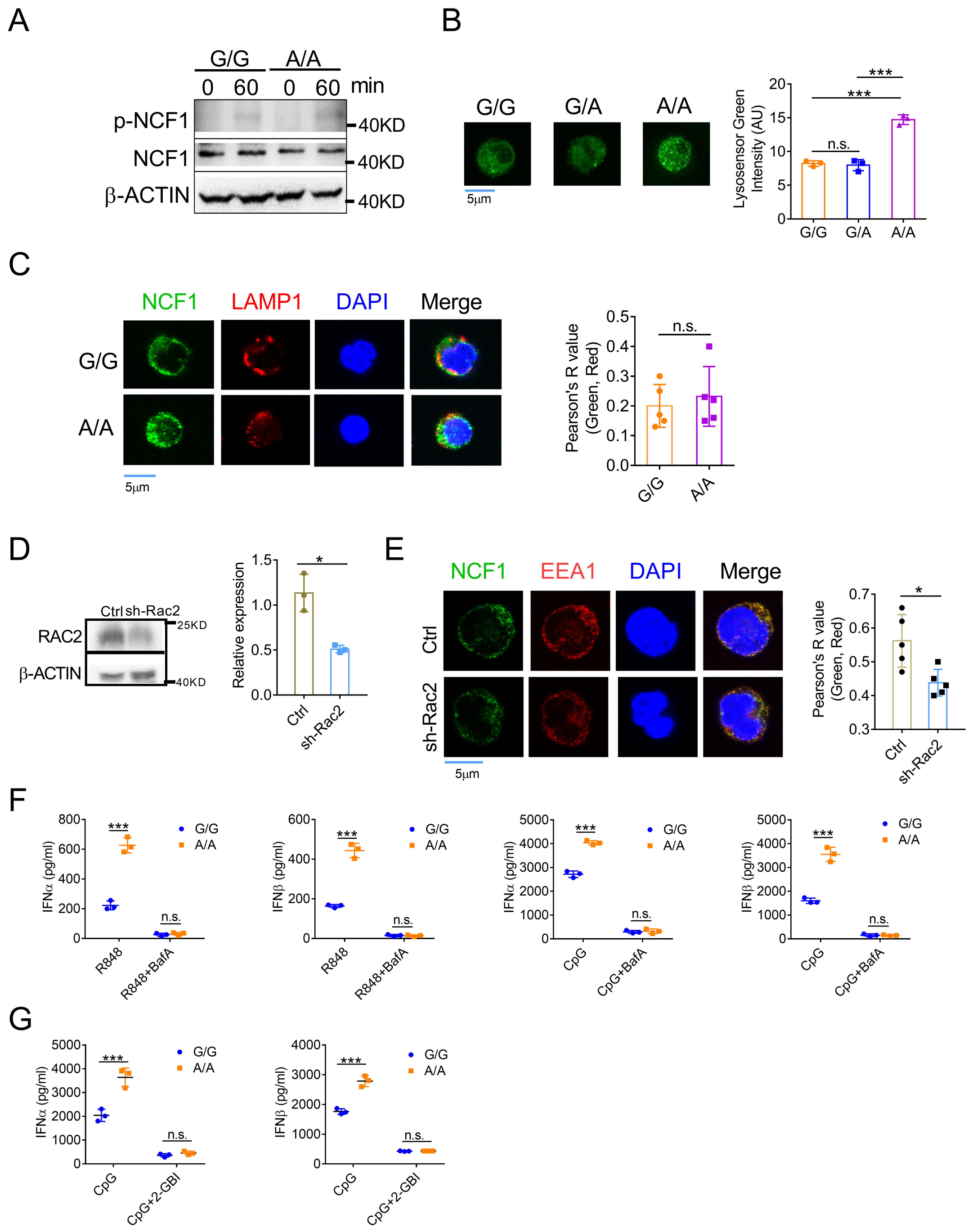

Supplemental Figure 4. The mechanism of NCF1 p.R90H variation on the activation of pDCs. (A-C) G/G, G/A and A/A pDCs generated by FLT3L induction in vitro were stimulated with CpG for 1 hour. (A) The levels of phospho-NCF1 (p-NCF1) and NCF1 were detected. β-ACTIN was used as loading control. (B) The pH of late endosome/lysosome was indicated by lysosome sensor, detected by con-focal, and then measured by Image J. One point indicates mean of Pearson's R values from 10 cells and total of 30 cells per group were calculated. Scale bar represents 5 μm. p values were determined by one-way ANOVA with Tukey's multiple comparison test. (C) The co-localization of NCF1 (green) and LAMP1 (Red) was detected by confocal microscopy and analyzed by Image J. (D, E) Lineage$^-$c-kit^{hi} bone marrow progenitors

were isolated from WT mice by using FACS. Lentivirus expressing shRNA specific targeting *Rac2* was transduced into bone marrow progenitors. Cells were cultured with 200ng/mL FLT3L for 8 days. Then pDCs were sorted and subjected to further study. (D) Western blot detection of RAC2. n=3. *p* values were determined by a two-tailed paired *t* test. (E) PDCs transfected with empty (Ctrl) or shRNA lentivirus targeting *Rac2* (sh-Rac2) were stimulated with CpG for 2 hours. The co-localization of NCF1 (green) and EEA1 (Red) was detected by confocal microscopy and analyzed by Image J. One point indicates mean of Pearson's R values from 6 cells and total of 30 cells per group were calculated. Scale bar represents 5 μm. *p* values were determined by a one or two-tailed unpaired *t* test. Error bars represent SEM. (n.s. not significant). (F) PDCs were pre-treated with or without 100nM bafilomycin A (BafA) for 1 hour and then stimulated with 10μg/mL R848 or 0.5μM ODN2216 (CpG) for 24 hours. (G) PDCs were pre-treated with or without 2-GBI for 1 hour and then stimulated with 0.5μM ODN2216 (CpG) for 24 hours. The expression of type I IFNs in supernatant was detected by ELISA. n=3. Data were analyzed by using two-way ANOVA. Error bars represent SEM. (*p<0.05, ***p<0.001, n.s. not significant).

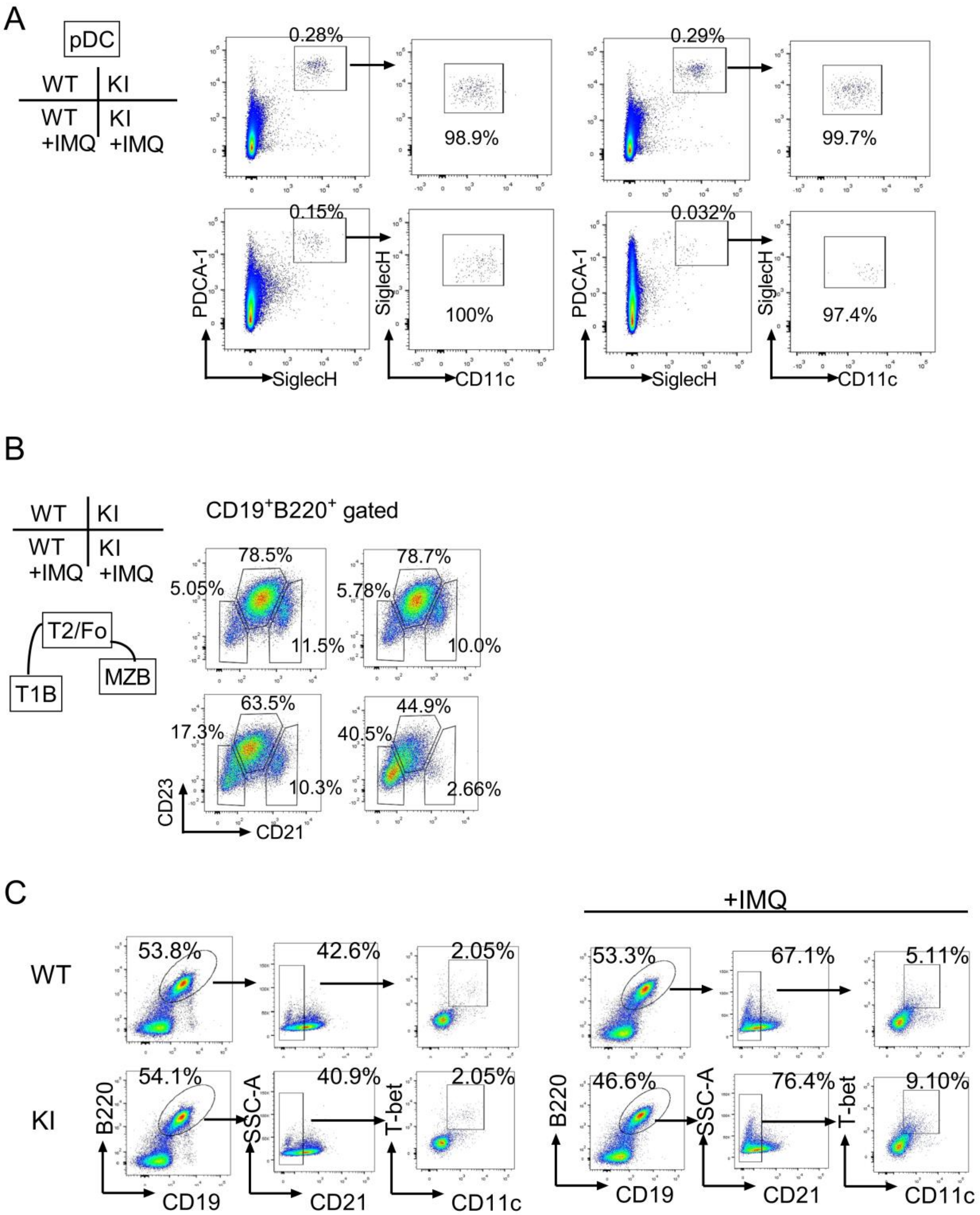

Supplemental Figure 5. NCF1 p.R90H facilitates the aberrant activation of pDC and B cells. The cellular gating strategies of (A) pDCs (CD11c^{int}, SiglecH^{+}, PDCA-1^{+}) and (B) B cells subsets, including MZB (CD19^{+}, B220^{+}, CD21^{hi}, CD23^{-}), T2/Fo B cells (CD19^{+}, B220^{+}, CD21^{lo}, CD23^{hi}), T1 B cells (CD19^{+}, B220^{+}, CD21^{-}, CD23^{-}) and (C) ABC cells (CD19^{+}, B220^{+}, CD21^{-}, CD11c$^{+,}$ T-bet^{+}) in Figure 4 are shown.

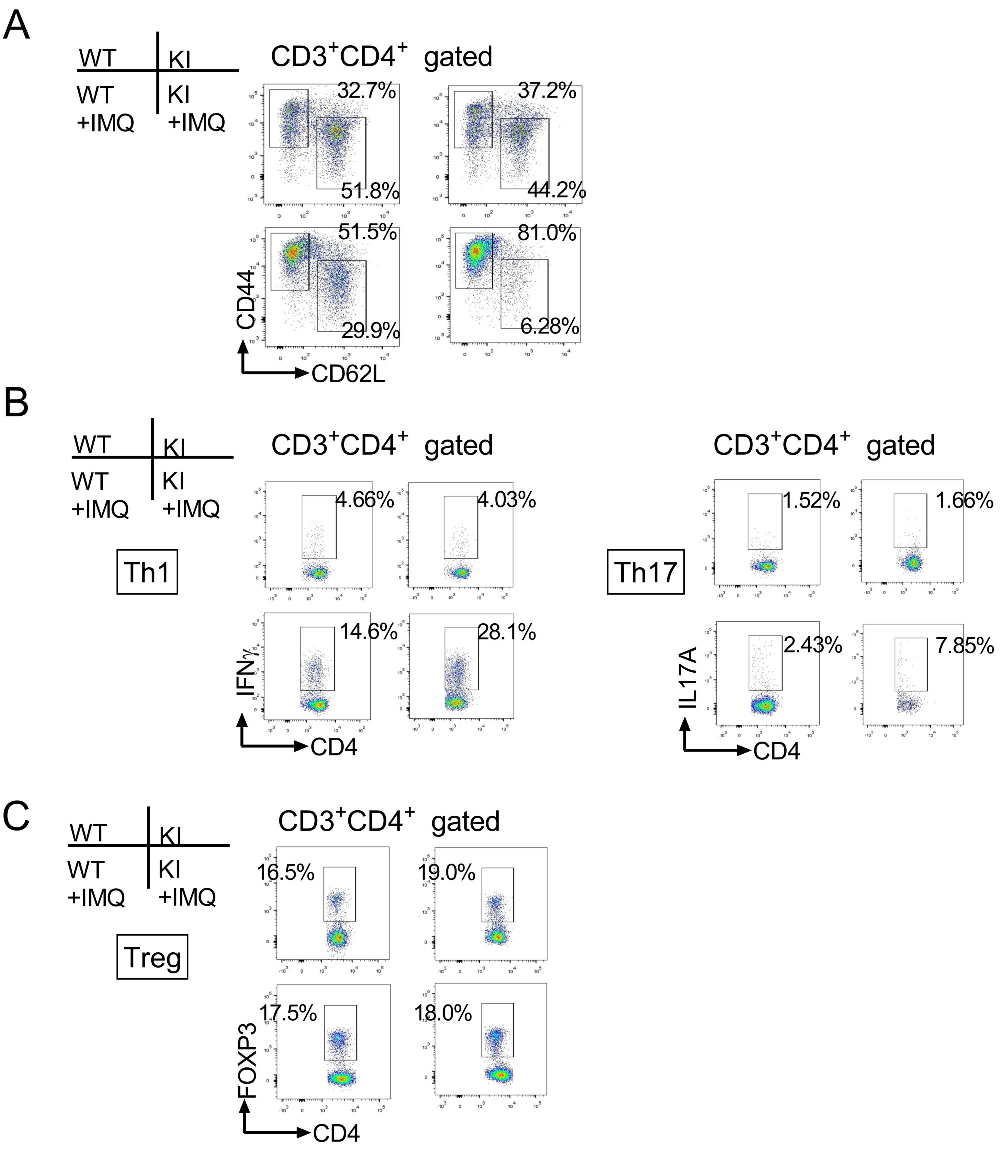

Supplemental Figure 6. NCF1 p.R90H facilitates the aberrant activation of T cells. (A) The FACS analysis of naïve T cells (CD3$^+$, CD4$^+$, CD44^{lo}, CD62L^{hi}), activated T cells (CD3$^+$, CD4$^+$, CD44^{hi}, CD62L^{lo}), (B) Th1 (CD3$^+$, CD4$^+$, IFNγ$^+$), Th17 (CD3$^+$, CD4$^+$, IL-17A$^+$) and (C) Treg (CD3$^+$, CD4$^+$, Foxp3$^+$) in Figure 4 are shown.

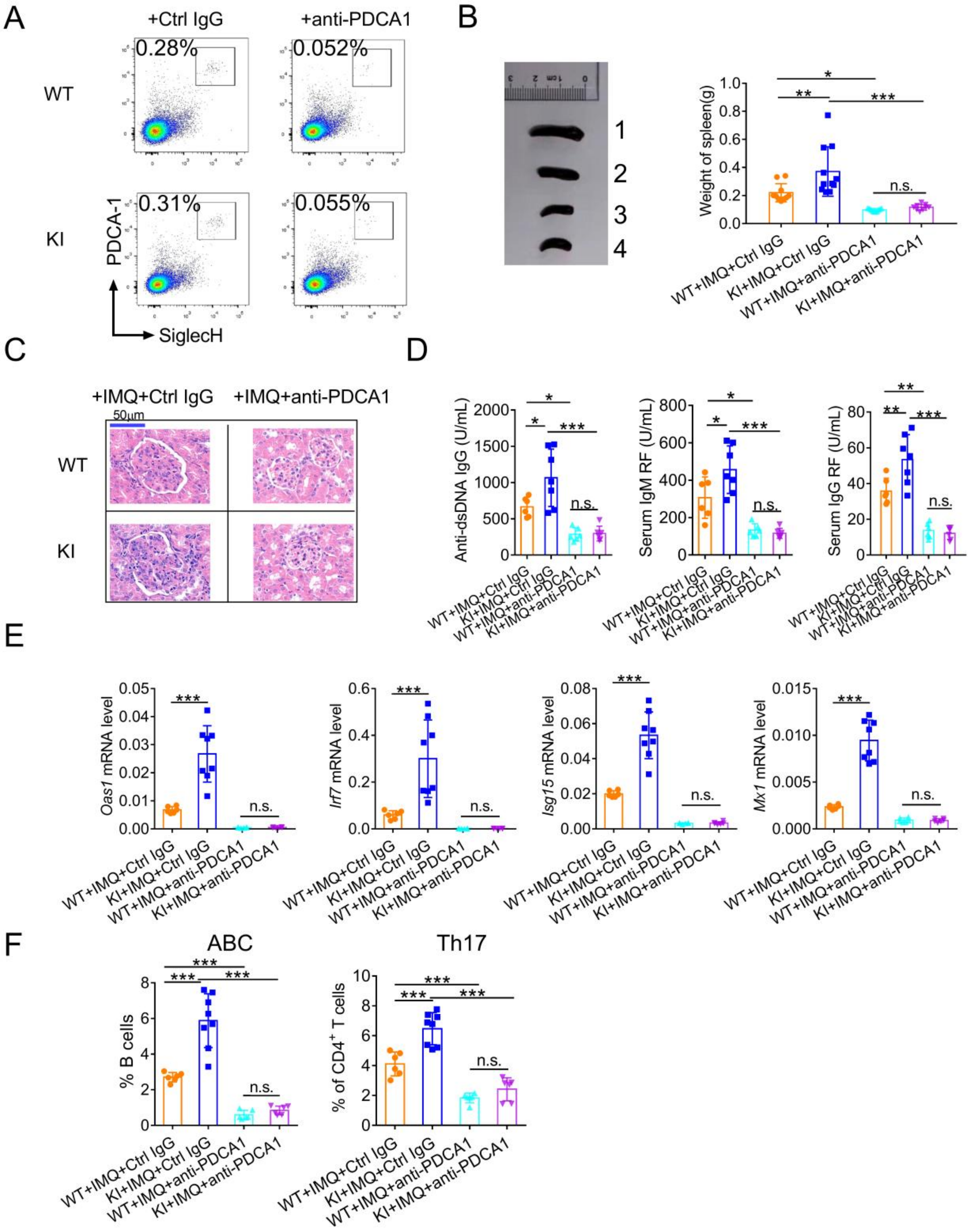

Supplemental Figure 7. PDC depletion eliminates the pathogenic aggravation caused by NCF1 p.R90H. (A) WT and KI mice were intraperitoneally pre-treated with anti-PDCA1 antibody (anti-PDCA1) or rat IgG2b isotype control (Ctrl IgG) 4 days before IMQ application. The efficiency of pDC depletion. (B-F) Mice were then treated with IMQ, together with Ctrl IgG or anti-PDCA1 antibodies for 8 weeks. (B) The picture and the weight of spleen. n=9-11. 1, WT+IMQ+Ctrl IgG; 2, KI+IMQ+Ctrl IgG; 3, WT+IMQ+anti-PDCA1; 4, KI+IMQ+anti-PDCA1. (C) H&E staining of kidney. (D) The levels of anti-dsDNA antibodies, serum IgM RF and IgG RF. (E) The expression of *Oas1*, *Irf7*, *Isg15* and *Mx1* in the kidney of mice. (F) FACS analysis of ABCs and Th17 cells in the spleen. n=6-8. Data were analyzed by using

one-way ANOVA with Tukey's multiple comparison test. Error bars represent SEM. (*$p<0.05$, **$p<0.01$, ***$p<0.001$, n.s. not significant).

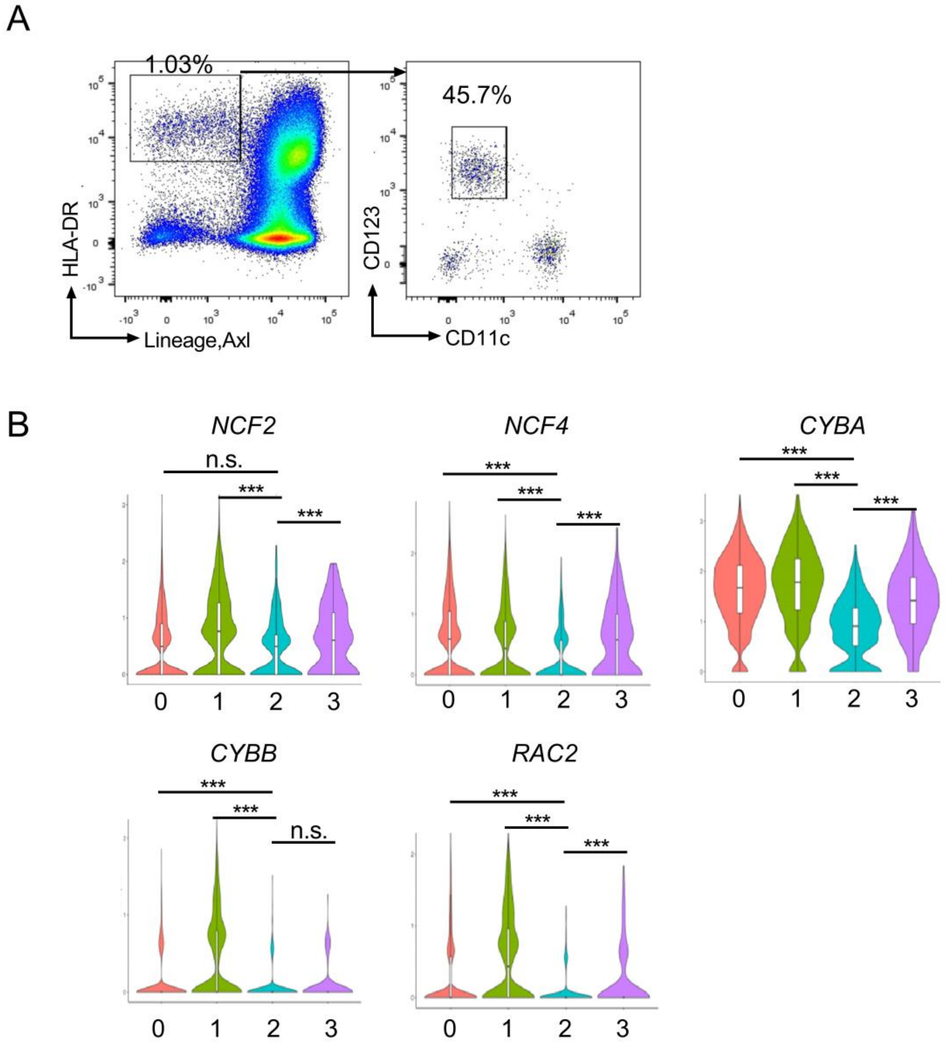

Supplemental Figure 8. Sorting strategy of human primary pDCs. (A) PDCs were sorted as Lineage$^-$ (CD3/CD14/CD16/CD19/CD20/CD56), HLA-DR$^+$, CD11c$^-$, AXL$^-$, CD123$^+$. The purity of pDCs was >99.9%. (B) The expression of *NCF2*, *NCF4*, *CYBA*, *CYBB* and *RAC2* in pDC subpopulations. *p* values were determined by using Wilcoxon test. (***p<0.001).

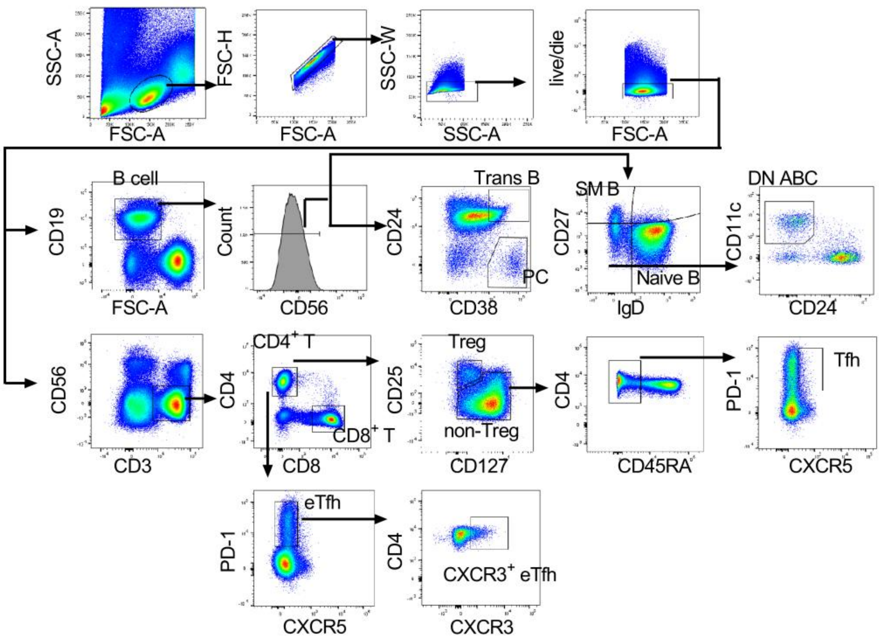

Supplemental Figure 9. Gating strategies of immune cells subsets from SLE patients. Samples in Figure 6 were analyzed by FACS using indicated markers.

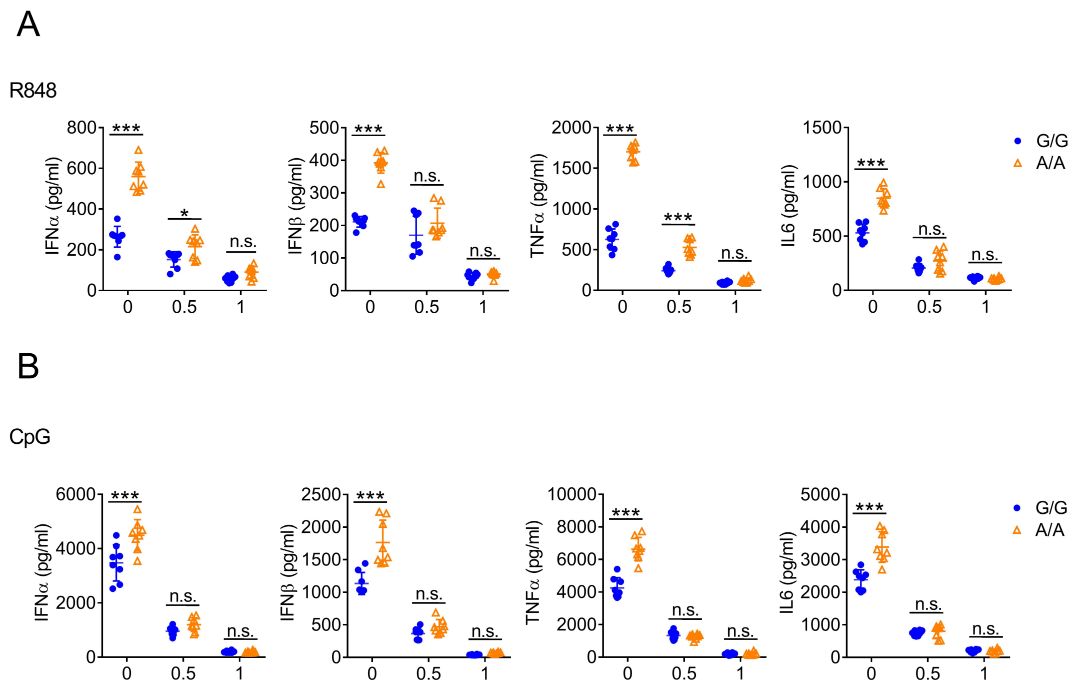

Supplemental Figure 10. HCQ alleviates the excessive activation of pDCs resulted from NCF1 p.R90H. (A, B) Splenic pDCs were pre-treated with 0, 0.5 or 1 μM HCQ for 2 hours, and then stimulated with R848 or CpG for 24 hours. The expression of IFNα, IFNβ, TNFα and IL6 was detected. n=8. *p* values were determined by two-way ANOVA. Error bars represent SEM. (*p<0.05, **p<0.01, ***p<0.001, n.s. not significant).

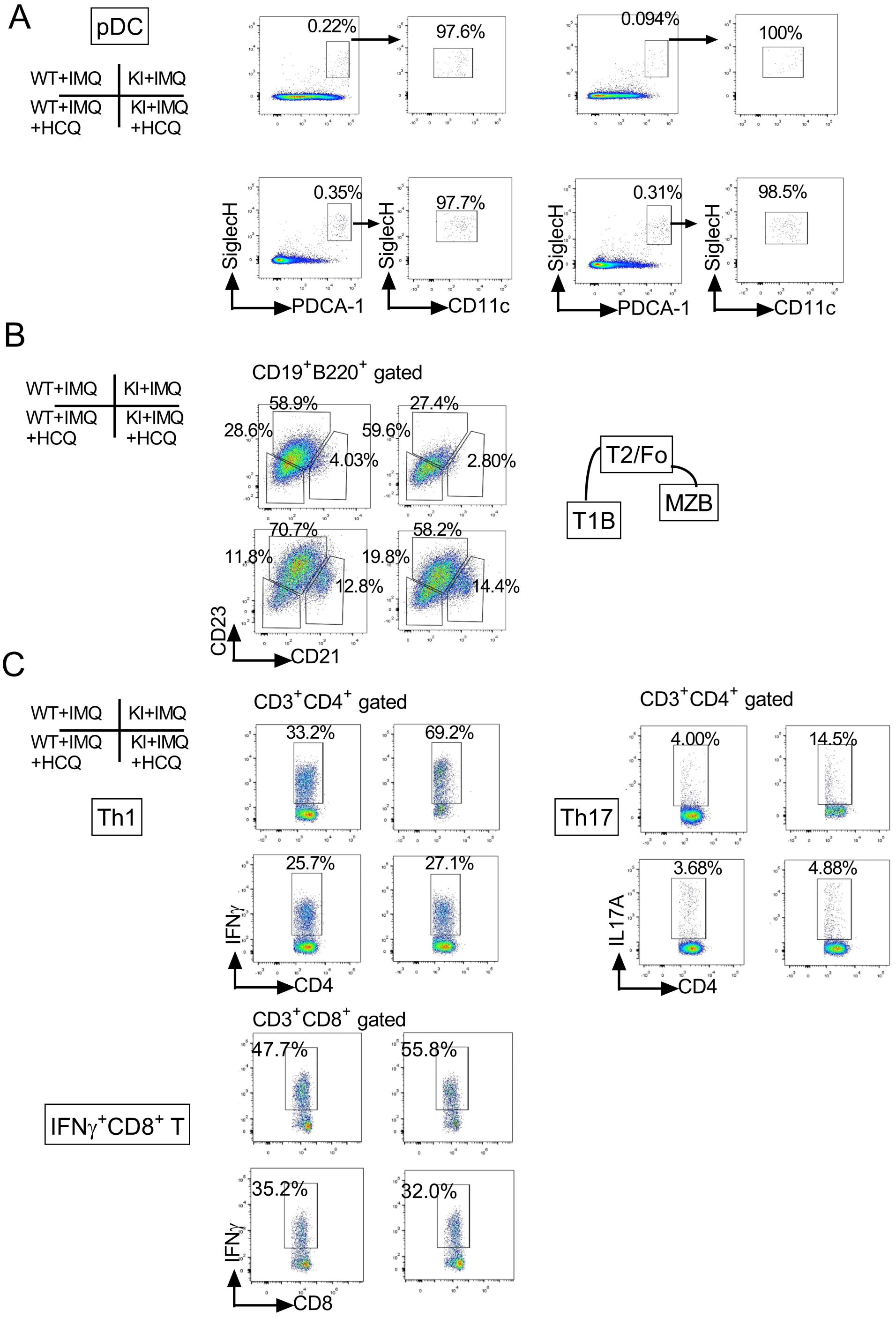

Supplemental Figure 11. FACS analysis of immune cells suppressed by HCQ. The FACS gating strategies of (A) pDCs (CD11c^{int}, SiglecH^{+}, PDCA-1^{+}), (B) B cells subsets, including MZB (CD19^{+}, B220^{+}, CD21^{hi}, CD23^{-}), T1 B (CD19^{+}, B220^{+}, CD21^{-}, CD23^{-}) and T2/Fo B cells (CD19^{+}, B220^{+}, CD21^{lo}, CD23^{hi}), and (C) T cell subsets, including Th1 (CD3^{+}, CD4^{+}, IFNγ^{+}), Th17 (CD3^{+}, CD4^{+}, IL17A^{+}) and IFNγ^{+} CD8^{+} T cells (CD3^{+}, CD8^{+}, IFNγ^{+}) in Figure 8 are shown.

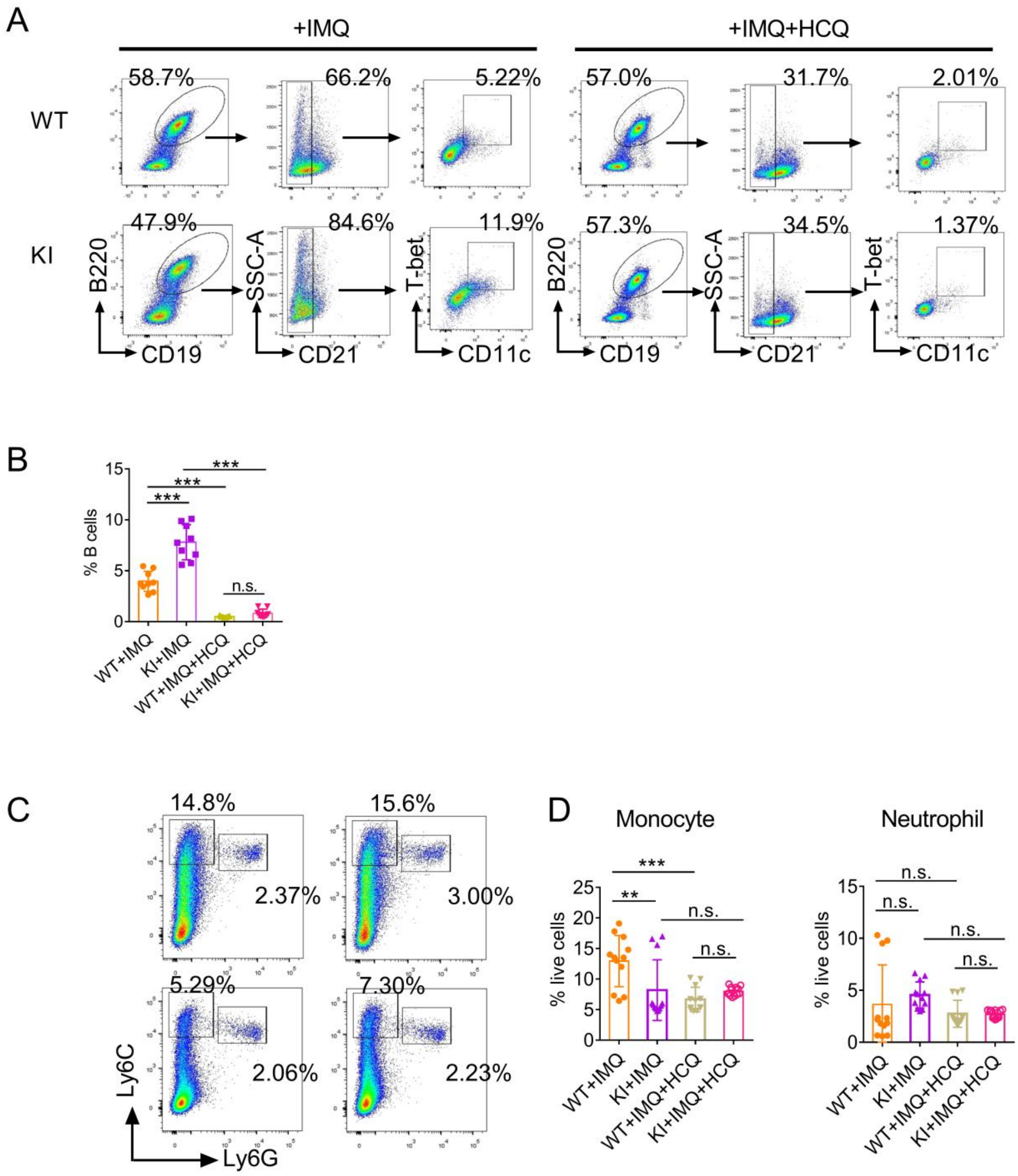

Supplemental Figure 12. Impact of NCF1 p.R90H and HCQ on ABCs, monocytes and neutrophils. WT and KI mice were treated with IMQ, together with or without HCQ for 8 weeks. Splenocytes were isolated and analyzed by FACS. (A) Gating strategy of ABCs (CD19$^+$, B220$^+$, CD21$^-$, CD11c$^+$, T-bet$^+$). n=9. (B) The proportion of ABCs. (C) FACS analysis of monocytes (Ly6C^{hi}, Ly6G$^-$) and neutrophils (Ly6C^{lo}, Ly6G$^+$). (D) Statistics of monocytes and neutrophils. p values were determined by one-way ANOVA with Tukey's multiple comparison test. Error bars represent SEM. (**p<0.01, ***p<0.001, n.s. not significant).

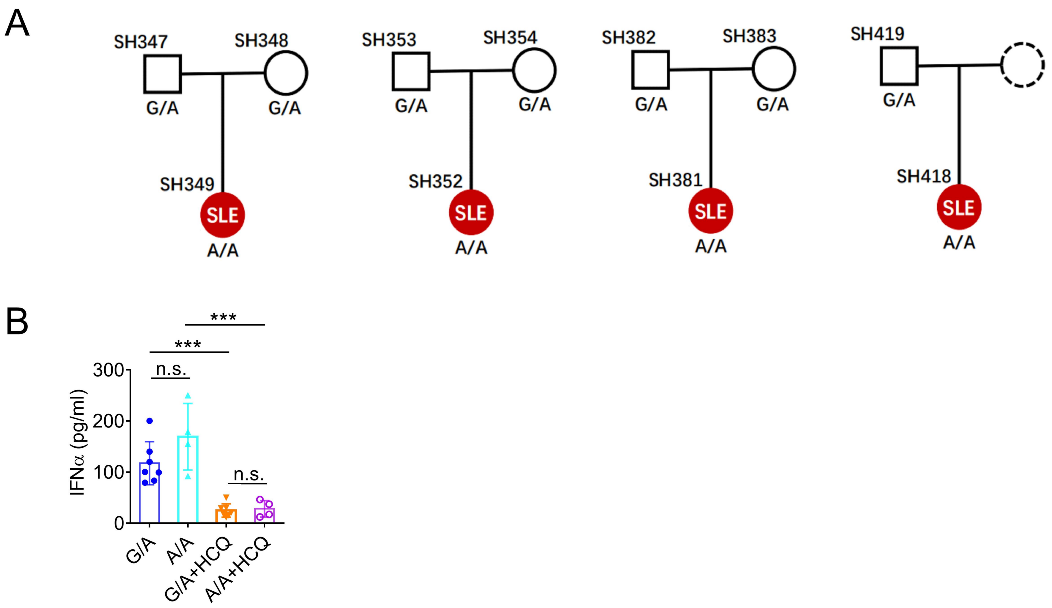

Supplemental Figure 13. The therapeutic effect of HCQ on PBMCs from SLE patients with NCF1 p.R90H. (A) The genetic map of SLE families. (B) PBMCs were pre-incubated with 0.5μM HCQ for 2 hours and stimulated with CpG for 24 hours. The expression of IFNα was detected. SH indicates sample number. *p* values were determined by one-way ANOVA with Tukey's multiple comparison test. Error bars represent SEM. (***p<0.001, n.s. not significant).

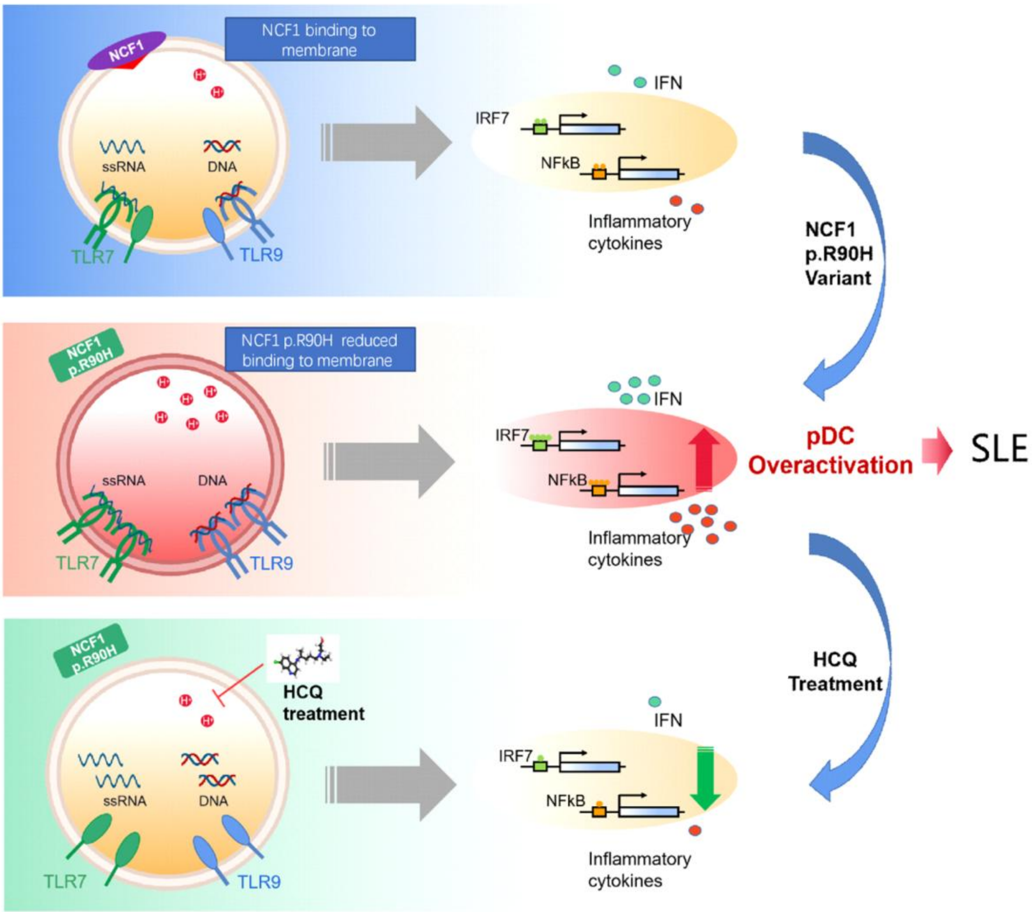

Supplemental Figure 14. Regulation model. NCF1 p.R90H variant leads to impaired localization of NCF1 on the endosomal membrane, followed by acidified pH and more cleaved activation of TLR receptors, which facilitates pDC activation and lupus progression. HCQ application alleviates the aggravation of NCF1 p.R90H on lupus, and this SNP could serve as a genetic biomarker for HCQ treatment.

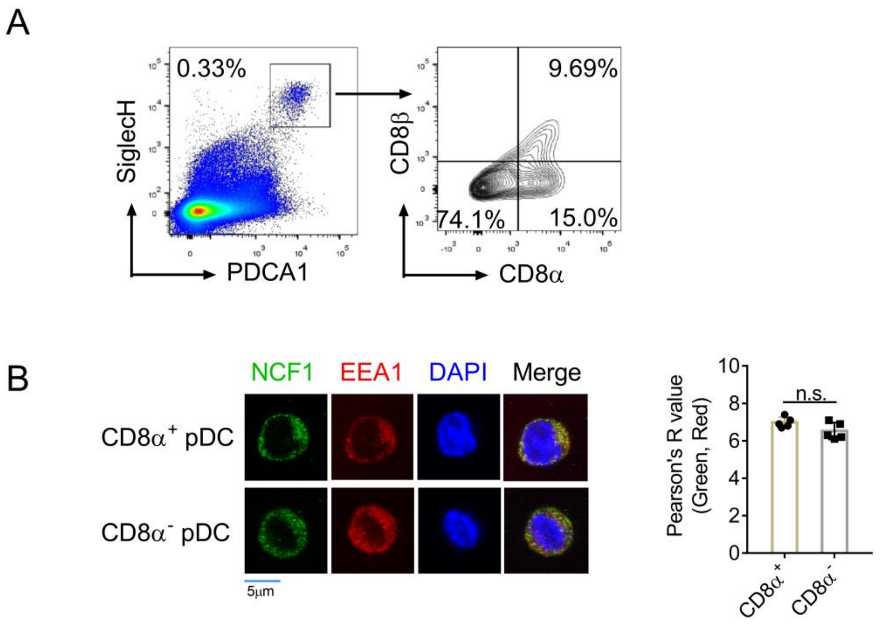

Supplemental Figure 15. CD8α$^-$ and CD8α$^+$ pDCs show similar endosomal localization of NCF1. Splenic pDCs subsets were isolated and stimulated with 10μg/mL R848 for 1 hour. The colocalization of NCF1 and EEA1 was detected by fluorescence microscope. (A) The gating strategy of pDC subsets. (B) The co-localization of NCF1 (green) and EEA1 (Red) was detected by confocal microscopy and analyzed by Image J. One point indicates mean of Pearson's R values from 6 cells and total of 30 cells per group were calculated. Scale bar represents 5 μm. *p* value was determined by a two-tailed unpaired *t* test. Error bars represent SEM. (n.s. not significant).

Supplemental Tables

Supplemental Table 1. Demographic and clinical characteristics of the patients.

	SLE patients
Number	49
Age (years), mean ± SD	35.4 ± 10.5
Female, n (%)	49 (100%)
Disease Duration (months), median (IQR) *	53.8 (105)
**System involvement, n (%) * ¶**	
Mucocutaneous	12 (30.0%)
Musculoskeletal	3 (7.5%)
Neuropsychiatric	1 (2.5%)
Cardiorespiratory	4 (10.0%)
Renal	19 (47.5%)
Hematological	16 (40.0%)
**Disease assessment, median (IQR) ***	
SLEDAI	7.0 (6.0)
**Laboratory tests**	
High ESR, (positive/tested)	24/38
High CRP, (positive/tested)	10/20
Low C3, (positive/tested)	30/40
ANA, (positive/tested)	37/38

Anti-Smith antibody, (positive/tested)	4/38
Anti-RNP antibody, (positive/tested)	14/38
Anti-dsDNA (IU/mL), median (IQR) *	26.2 (41.0)
**Treatment, n (%) [#]**	
Prednisone	37 (100%)
Hydroxychloroquine	22 (59.5%)
Immunosuppressants	14 (37.8%)

SLEDAI Systemic Lupus Erythematosus Disease Activity Index, *ESR* Erythrocyte sedimentation rate, *CRP* C-Reactive Protein, *C3* complement 3, *ANA* anti-nuclear antibody, *RNP* ribonucleoprotein.

* Among the 49 patients, 9 patients' clinical information were missing. The percentage calculation was based on 40 patients. [#] 12 patients' treatment information were missing. The percentage calculation was based on 37 patients. ¶ System involvement indicated all the system involvements during the disease course of the patients.

Supplemental Table 2. SLE families information.

		23			24			35			47	
sample ID		SH349	SH350	SH351	SH352	SH353	SH354	SH381	SH382	SH383	SH418	SH419
gender		Female	Male	Female	Female	Male	Female	Female	Male	Female	Female	Male
patient_age		6	37	34	11			14	42	37	13	35
relationship		Patient	Father of SH349	Mother of SH349	Patient	Father of SH352	Mother of SH352	Patient	Father of SH381	Mother of SH381	Patient	Father of SH418
Autoimmune disease	SLE	y	n	n	y	n	n	y	n	n	y	n
	Hashimoto's Thyroiditis	n			n			n			n	
	Grave's disease	n			n			n			n	
	T1D	n			n			n			n	
	Vitiligo	n			n			n			n	
	Alopecia	n			n			n			n	
	RA	n			n			n			n	
	Sjogren's Sd	n			n			n			n	
	Vasculitis	n			n			n			n	
	Other											
Lupus ACR	Acute Cutaneous lupus	y			y			y			y	

criteria	Chronic cutaneous lupus											
	Aphthous ulcers	n			y			y			y	
	Alopecia	n			n			n			n	
	Arthritis (2 or more joints)	y			y			y			n	
	Serositis	n			n			n			n	
	Renal: Red cell casts	n			y			n			y	
	Renal: >500g protein/24h	n			n			n			y	
	Neurological: Seizures	n			n			n			n	
	Neurological: Psychosis	n			n			n			n	
	Neurological: myelitis	n			n			n			n	
	Autoimmune hemolytic anemia	n			n			n			n	
	Leukopenia:<4.0 ×10^9/L	n			n			n			n	
	Lymphopenia:<1.0 ×10^9/L	n			n			y			n	
	Thrombocytopenia:<1.0 ×10^9/L	n			n			n			n	
	ANA	1：640			1:160			1: 320				
	dsDNA antibodies (IU/ml)	8.4 IU/ML			28.94 IU/ML			65 IU/ML			45.98 IU/ML	
	Sm	n			n			y				

	Lupus anticoagulant	n			n			y				
	Cardiolipin antibodies (medium or high titre)	n			n			n				
	beta-2 glycoprotein I antibodies	n			n			n				
	Direct Coombs Test	n			n			n				
	Low C3	n			y			y			y	
	Low C4	n			n			y			y	
Additional lupus phenotypes: a Vascular	Raynaud's phenomenon	n			n			n			n	
	Digital vasculitis	n			n			n			n	
	Cutaneous vasculitis	n			n			n			n	
	Arterial thromosis	n			n			n			n	
	Deep venous thrombosis	n			n			n			n	
	Acute pulmonary embolus	n			n			n			n	
Additional lupus phenotypes: b Renal	Renal	n			n			n			y	
	WHO Glomerulonephritis histological class											
	Nephrotic symdrome	n			n			n			n	
	Highest serum creatinine (umol/L)	24 umol/L			58 umol/L			30 umol/L			34 umol/L	
	Lowest GFR											

Additional lupus phenotypes: c Sicca symptoms	Dry mouth	y			y			y			y	
	Dry eyes	n			n			n			n	
Additional lupus phenotypes: d Musculoskeletal	Tenosynovitis	n			n			n			n	
	Joint subluxation	n			n			y			n	
	Myositis	n			n			n			n	
Additional lupus phenotypes: e Gastrointestinal	Hepatitis	n			n			n			n	
	Mesenteric ischaemia	n			n			n			n	
	Pacreatitis	n			n			n			n	
Additional lupus phenotypes: g Serology	RNP	n			y			y			n	
	SSA	n			n			y			n	
	SSB	n			n			y			n	
	Ribosomal P	n			n			n			n	
	Smooth muscle	n			n			n				
	Thyroidperoxidase	n			n			n				

	Thyroglobulin	n			n			n				
	Gastric parietal cell	n			n			n				
	Skin	n			n			y			y	
	Hypergammaglobulinaemia	n			n			n				
	Other											

“y” = Yes, “n” = No.

Supplemental Table 3. List of antibodies and reagents

Murine antibodies

Antibodies	Source	Cat#
Anti-mouse PDCA1-BV421	BioLegend	127023
Anti-mouse CD4-FITC	BioLegend	100406
Anti-mouse CD21-APC	BioLegend	123412
Anti-mouse B220-PE-CY7	BioLegend	103222
Anti-mouse MHCII-BV510	BioLegend	107636
Anti-mouse CD11c-percpcy5.5	BioLegend	136504
Anti-mouse CD62L-BV510	BioLegend	104441
Anti-mouse CD11c-FITC	BioLegend	117306
Anti-mouse CD19-BV510	BioLegend	115546
Anti-mouse CD44-PE-CY7	BioLegend	103030
Anti-mouse CD23-FITC	BioLegend	101606
Anti-mouse CD3-APC	BioLegend	100236
Anti-mouse CD172a-APC	BioLegend	144014
Anti-mouse CD8α-Percp-cy5.5	BioLegend	100736
Anti-mouse SiglecH-PE	eBioscience	12-0333-82
Total IRF7 antibody	Santa Cruz	sc-74471
Anti-EEA1	Abcam	ab2900
Goat Anti-Rabbit IgG H&L (Alexa Fluor® 647)	Abcam	ab150083

Donkey polyclonal Secondary Antibody to Goat IgG - H&L (Alexa Fluor® 488)	Abcam	ab150129
Goat polyclonal to NCF1/p47-phox	Abcam	ab166930
Rabbit polyclonal to RAC2	Abcam	ab191527
Alexa Fluor® 647 anti-mouse CD107a (LAMP-1) Antibody	Biolegend	121610
Alexa Fluor® 647 Goat anti-mouse IgG (minimal x-reactivity) Antibody	Biolegend	405322
Anti-mouse IFNγ-APC	BD	562018
Anti-mouse IL-17A-PE	BD	561020
DAPI	BD	564907
β-Actin (13E5) Rabbit mAb	Cell Signaling Technology	#4970
Anti-rabbit IgG, HRP-linked Antibody	Cell Signaling Technology	#7074
Anti-mouse IgG, HRP-linked Antibody	Cell Signaling Technology	#7076
Phospho-IRF7 (Ser437/438) (D6M2I) Rabbit mAb	Cell Signaling Technology	#24129
NF-κB p65 (D14E12) XP® Rabbit mAb	Cell Signaling Technology	#8242
Phospho-NF-κB p65 (Ser536) (93H1) Rabbit mAb	Cell Signaling Technology	#3033
Catalase (D5N7V) Rabbit mAb	Cell Signaling Technology	#14097
Anti-TLR7	Novus	NBP2-24906
Anti-TLR9	Novus	NBP2-24729
Phospho-p47phox (Ser370) Polyclonal Antibody	Thermo Fisher	PA5-36863

InVivoMAb anti-mouse CD317	Bio X Cell	BE0311
InVivoMAb rat IgG2b isotype control	Bio X Cell	BE0090

Human antibodies

**Antibodies**	**Source**	**Cat#**
Anti-human CD19-BV650	BioLegend	302238
Anti-human CD38-BV605	BioLegend	303532
Anti-human IgD-BV510	BioLegend	348220
Anti-human CD8-pacific blue	BioLegend	301033
Anti-human PD1-PE-CF594	BioLegend	329940
Anti-human CXCR3-PE	BioLegend	353705
APC anti-human Lineage Cocktail (CD3, CD14, CD16, CD19, CD20, CD56)	Biolegend	348803
Anti-human CD24-BV711	BD	563401
Anti-human CD11c-BUV395	BD	563787
Anti-human CD3-BV786	BD	563799
Anti-human CD4-BUV496	BD	564651
Anti-human CXCR5-AF647	BD	558113
Anti-human CD25-APC-R700	BD	565106
Anti-human CD127-BB700	BD	566398
Anti-human CD56-BUV737	BD	564447

Anti-human CD123-PECY7	BD	560826
Anti-human CD11c-percpcy5.5	BD	565227
Anti-human HLA-DR-FITC	BD	555811
Anti-human AXL-APC	R&D	FAB154A
Anti-human CD27-APC-EF780	eBioscience	47-0279
Anti-human CD45RA-PE-CY7	eBioscience	25-0458-73
Fc block	BioLegend	422301
LIVE/DEAD® Stain Kit Green Fluorescent	Invitrogen	L23101

Reagents and kits

**Reagents**	**Source**	**Cat#**
2-Deoxy-D-glucose (2-DG)	Selleck Chemical	S4701
Acetylcysteine (N-acetylcysteine)	Selleck Chemical	S1623
Etomoxir sodium salt	Selleck Chemical	S8244
Metformin HCl	Selleck Chemical	S1950
Hydroxychloroquine Sulfate	Selleck Chemical	S4430
Bafilomycin A1	Selleck Chemical	S1423
2-Guanidinobenzimidazole	Sigma	G11802
Cell Staining Buffer	BioLegend	420210
Recombinant Murine Flt3-Ligand	Peprotech	250-31L
Mouse IFN-alpha ELISA Kit	R&D	42120

Human IFN-alpha ELISA Kit	R&D	41100
Mouse IFN-beta ELISA Kit	R&D	42400
ELISA MAX™ Deluxe Set Mouse IL6	Biolegend	431304
ELISA MAX™ Deluxe Set Mouse TNFα	Biolegend	430904
R848 (Resiquimod)	Invivogen	tlrl-r848
ODN 2216	Invivogen	tlrl-2216
PtdIns-(3,4)-P2 (1,2-dihexanoyl) (sodium salt)	Cayman Chemical	10007759
1-Palmitoyl-3-oleoyl-sn-glycero-2-PE	Cayman Chemical	15104
1-Palmitoyl-2-oleoyl-sn-glycero-3-PC	Cayman Chemical	15102
LBIS Mouse IgG Rheumatoid Factor ELISA Kit	FUJIFILM Wako<br>Shibayagi Corporation	637-02679
LBIS Mouse IgM Rheumatoid Factor ELISA Kit	FUJIFILM Wako<br>Shibayagi Corporation	634-02689
LBIS Mouse anti-dsDNA ELISA Kit	FUJIFILM Wako<br>Shibayagi Corporation	631-02699
OxyBURST™ Green H2DCFDA	Thermo Fisher	D2935
LysoSensor™ Green DND-189	Thermo Fisher	L7535
TaqMan™ Genotyping Master Mix	Thermo Fisher	4371353
MitoSOX™ Red	Thermo Fisher	M36008
SuperBlock™ (TBS) Blocking Buffer	Thermo Fisher	37535
Anti-Mouse/Rat Foxp3 Staining Set PE	eBioscience	72-5775-40

BD™ Cytometric Bead Array (CBA) Mouse TNF Flex Set	BD	558299
BD™ Cytometric Bead Array (CBA) Mouse IL6 Flex Set	BD	558301
Fixation/Permeabilization Solution Kit with BD GolgiStop™	BD	554715
Long Range PCR kit	Qiagen	206402
Diamond Plasmacytoid Dendritic Cell Isolation Kit II, human	Miltenyi Biotech	130-097-240
pLKO.1-shRac2-puro-CMV-tGFP lentivirus	Sigma	TRCN0000065343

Supplemental Table 4. Primers used in this study.

**Genes**	**Forward primer (5’-3’)**	**Reverse primer (5’-3’)**
mouse mx1	CTGAGGGCTCTGGGTGT	GTAACAATACCACTGCCTCTG
mouse irf7	CCTGATCCTGGTGAAGCTGG	TGGGAGTTGGGATTCTGAGTC
mouse oas1	TTTGAGCAGGTAGAAGAGAACT	GCATCAGAAGCACGGAGT
mouse isg15	AGAGCAAGCAGCCAGAAG	CACCGTCATGGAGTTAGTCAC
mouse ifit1	ATGGGAGAGAATGCTGATGG	AGGAACTGGACCTGCTCTGA
mouse β-actin	ATGCTCCCCGGGCTGTAT	CATAGGAGTCCTTCTGACCCATTC
mouse Catalase clone	GGAATTCATGTCGGACAGTCGGGACC	GGCGGCCGCTTACAGGTTAGCTTTTCCCTTC
human mx1	GGGTAGCCACTGGACTGA	AGGTGGAGCGATTCTGAG
human ifit1	GCCTCCTTGGGTTCGTCTACAA	TCAAAGTCAGCAGCCAGTCTCA
human oas1	GAAGGCAGCTCACGAAAC	TTCTTAAAGCATGGGTAATTC
human irf7	TGAAGCTGGAACCCTGG	GATGTCGTCATAGAGGCTGTT
Human rpl13a	CCTGGAGGAGAAGAGGAAAGAGA	TTGAGGACCTCTGTGTATTTGTCAA

Taqman assay for *NCF1* genotyping

	Forward primer	Reverse primer	Reporter 1 Sequence	Reporter 2 Sequence
NCF1 p. R90H	CAGCTCCCAAGTGGTTTGAC	GGTGGGCAGGCTCATGA	CCTGGCGGTTCTC	CCTGGTGGTTCTC
